# Measuring the suicidal mind: The ‘open source’ Suicidality Scale, for adolescents and adults

**DOI:** 10.1371/journal.pone.0282009

**Published:** 2023-02-23

**Authors:** Keith M. Harris, Lu Wang, Guanglun M. Mu, Yanxia Lu, Cheryl So, Wei Zhang, Jing Ma, Kefei Liu, Wei Wang, Melvyn Wei-bin Zhang, Roger C. Ho

**Affiliations:** 1 School of Psychology, Charles Sturt University, Bathurst, New South Wales, Australia; 2 School of Psychology, University of Queensland, St Lucia, Queensland, Australia; 3 School of Environmental and Life Sciences, University of Newcastle, Callaghan, New South Wales, Australia; 4 Education Futures, University of South Australia, Adelaide, South Australia, Australia; 5 Department of Medical Psychology and Ethics, School of Basic Medical Sciences, Shandong University, Jinan, Shandong, China; 6 Private Clinician, Hong Kong; 7 School of Medicine and Health Management, Huazhong University of Science and Technology, Hubei, Wuhan, China; 8 School of Politics and Public Administration, Zhengzhou University, Zhengzhou, Henan, China; 9 Yale School of Medicine, Yale University, New Haven, Connecticut, United States of America; 10 Department of Psychology, Norwegian University of Science and Technology, Trondheim, Norway; 11 Biomedical Institute for Global Health Research and Technology, National University of Singapore, Singapore, Singapore; 12 Department of Psychological Medicine, National University of Singapore, Singapore, Singapore; Brown University Warren Alpert Medical School, UNITED STATES

## Abstract

Clinicians are expected to provide accurate and useful mental health assessments, sometimes in emergency settings. The most urgent challenge may be in calculating suicide risk. Unfortunately, existing instruments often fail to meet requirements. To address this situation, we used a sustainable scale development approach to create a publicly available Suicidality Scale (SS). Following a critical review of current measures, community input, and panel discussions, an international item pool survey included 5,115 English-speaking participants aged 13–82 years. Revisions were tested with two follow-up cross-sectional surveys (*N*s = 814 and 626). Pool items and SS versions were critically examined through item response theory, hierarchical cluster, factor and bifactor analyses, resulting in a unidimensional eight-item scale. Psychometric properties were high (loadings > .77; discrimination > 2.2; test-retest *r* = .87; internal consistency, ω = .96). Invariance checks were satisfied for age, gender, ethnicity, rural/urban residence, first language, self-reported psychiatric diagnosis and suicide attempt history. The SS showed stronger psychometric properties, and significant differences in bivariate associations with depressive symptoms, compared with included suicide measures. The ‘open source’ Suicidality Scale represents a significant step forward in accurate assessment for people aged 13+, and diverse populations. This study provides an example of sustainable scale development utilizing community input, emphasis on strong psychometric evidence from diverse samples, and a free-to-use license allowing instrument revisions. These methods can be used to develop a wide variety of psychosocial instruments that can benefit clinicians, researchers, and the public.

## Introduction

Suicide resides at the very core of deaths by despair [1, 2]. Due to this importance, there are long-standing recommendations for clinicians to conduct routine suicide risk assessments [SRA; 3, 4]. However, low-validity SRAs can lead to poorly guided clinical decisions. As with other psychosocial constructs, quantifying the latent trait, suicidality, requires high instrument precision with a focus on the fundamental nature of the construct. Despite serious consequences, those selecting and using tests may not be giving sufficient attention to psychological science, particularly psychometrics [5–11]. A lack of focus on psychometric validity, and concerns over psychological science replication [12], has resulted in continued use of popular measures, regardless of demonstrated weaknesses.

In response to current assessment practices, a growing number of psychological scientists are advocating for greater emphasis on measurement validity over consistency [e.g., 13, 14]. That may be particularly relevant for SRAs, which have not notably improved since Beck and colleagues published the Scale for Suicide Ideation [SSI; 15] in 1979. To address the urgent need for accurate assessments, this study utilized a sustainable scale development approach for the Creative Commons licensed (free culture) Suicidality Scale (SS) for adolescents, adults, and diverse populations.

We hypothesize that a highly valid measure of the latent trait, current suicidality, may be the best candidate for predicting future suicidal distress and suicide. To measure a latent trait, we first need to define it and determine how it can be quantified. Many find the term suicidality useful as it encompasses the totality of the multifaceted suicidal mind. Decades of evidence and theory reveal a complicated dynamic of affective, cognitive, and behavioral attributes that are volatile but can also pose long-term risk [15–17]. We consider suicidality as the extant summation of one’s feelings, thoughts and behaviors related to taking one’s life. Facets which require strong empirical evidence if they are to form a highly accurate measure.

### Measurement models

To understand the current underwhelming state of SRAs we can look to the overwhelming popularity of classical test theory (CTT). There are various measurement models to consider when validating a latent trait instrument. The parallel model stipulates all items are equal in measuring the same trait with the same level of accuracy, identical response sets, and identical error [18, 19]. Similarly, CTT assumes a tau-equivalent model, identical to the parallel model but item errors may vary. The congeneric model, in contrast, assumes items measure the same latent trait but can vary in precision, response sets, and error. Also of importance, sum scores (summing item scores for a scale total) require tau-equivalence, all items and response steps are equally and uniformly quantifiable [20, 21]. Popular psychometric analyses such as Cronbach’s alpha, confirmatory factor analysis (CFA), receiver operating characteristics (ROC) and area under the curve (AUC), assume tau-equivalence [22, 23]. ROC also requires true binary outcomes [24]. The congeneric model, however, fits best with decades of evidence of a lack of SRA tau-equivalence, demonstrated through heterogeneity in factor loadings, discrimination, information functions, etc. [25–28]. Psychometric analyses consistent with congeneric models include factor analysis (FA), bifactor analysis (BA), McDonald’s omega, and IRT (item response theory).

Another fundamental decision is defining a measurement model as reflective or formative. Most psychological measures are reflective, highly correlated items that are indirect indicators of common factors. However, with formative measures, items may be loosely correlated but are required components of a composite factor [29]. A classic example of a formative factor is socioeconomic status, which can be derived through items on income, education, and other components. Many popular SRAs are formative, constructed through indexes–checklists of items that can be scored present/absent. The SAD PERSONS and Manchester Self-Harm Rule are indexes that are hypothesized to form cumulative values of suicide risk [30]. Many hospitals, clinicians and researchers use SRAs that include variables such as sex (males scored at-risk), relationship status (unpartnered scored at-risk), and major depressive disorder diagnosis (scored at-risk). Additionally, the DSM-V mood disorders group was reportedly developing an SRA index based on presence/absence of suicide attempts, plans, substance abuse, and living alone [31]. The implicit measurement hypothesis of SRA indexes is–population suicide risk and protective factors can be counted, and through simple addition and subtraction a highly accurate personal risk score can be calculated.

Despite decades of psychometric evidence and advances in statistical software, SRA validation studies have mostly used CTT methods, typically have not held instruments to high standards, and often included dichotomous items and outcomes. False dichotomies, for example, can violate core test assumptions, leading to false findings [32–34]. Nevertheless, WHO’s Composite International Diagnostic Interview [WMH-CIDI; 35], was tested using sum scores and ROC and AUC analyses, with disputable binary outcomes [36]. Many CIDI dichotomous items were integrated into the Columbia-Suicide Severity Rating Scale [C-SSRS; 37]. More recently, a moderate-sized computer-adaptive test study (CAT; *N* = 308) used 11 dichotomous and ordinal items, based partly on the C-SSRS, with factor loadings as low as .59 described as ‘strong’ [28]. The authors used sum scores and cutoffs to create opaque risk groups, with substantial trait overlap. These studies are consistent with most SRA validation efforts, measurement models are not specified, analyses may not be justified, and instruments fail to demonstrate strong validity.

The SSI, a decades-old standard for SRAs, was developed over years through modifying various instruments, resulting in 19 items with differing three-point response sets [15]. Developers used relevant psychological constructs (e.g., suicidal ideation, the wish to die) rather than demographics, pointing out that demographics can indicate group risk differences but are not appropriate for individual risk assessments. Numerous studies have examined SSI validity, but these have not led to significant improvements. For example, an IRT analysis [25] seemed to err on the side of consistency by concluding only two items should be revised or deleted. However, findings showed additional items with low discrimination (low ability in differentiating trait levels). Despite limitations, the SSI remains popular and is part of many test banks, including the PhenX Toolkit for genetic studies [38]. This has allowed for consistency between assessments, but at a cost to validity.

In addition to choosing the right model, there are other measurement details to consider. Pek and Flora [39] identified measurement problems as metrics (response sets, response options), and the construct (the closeness/distance between the observed scores and the hypothesized latent trait). To address such concerns, measurement validation should focus on the processes that produce changes in the instrument’s values [5]. For example, we could examine whether suicide attempt items can be adequately quantified through yes/no responses or whether a polytomous item including intent to die provides additional monotonic (increasing/decreasing) grades [40, 41]. That is, focusing on the underlying facets of an item that produce measurable changes when individuals move from low to higher risk.

### Study aims

The primary goal was to determine a unidimensional item set that would best capture the suicidal attributes that are most valid across diverse populations and ages. Suicidality was conceptualized as a latent trait composed of several interrelated facets. We chose a congeneric model [10, 20, 21], as relevant facets may be dimensional and are likely to vary on trait coverage and information captured. To achieve study aims, we employed theory-informed, but evidence driven scale development practices [e.g., 42–45]. Scale validity required evidence of strong model fit, high item discrimination and information levels, high predictive ability, and invariance by demographic groupings.

In addition, this study was aimed at providing an example of sustainable scale development, in support of the UN’s sustainable development goals [46]. For latent trait measures to be sustainable, they require very strong psychometrics and limited error. We aimed for community input, including review and suggestions on item wording. We also aimed for validity across diverse groups, as sustainable scales require demonstrated utility across demographics. Sustainable scales also need to be free to use and modifiable, so that low-income populations can use the instrument and future research can improve measurement accuracy.

## Materials and methods

### Transparency and openness

We utilized a multidisciplinary open science approach to help achieve our goals of contributing to sustainable development of good health and wellbeing, knowledge and skills sharing, and global partnerships. That includes making data, methods and analyses publicly available and making the SS freely available through a Creative Commons BY 4.0 license [47]. A preprint of an earlier version of this manuscript produced feedback, resulting in several modifications [48]. An open methods presentation provides additional information on study methods [49].

Survey participation was open to anyone meeting minimum age requirements with adequate language skills. All questions were voluntary, other than a mandatory minimum age gateway item. Forced-choice questions were not used as they can lead to higher dropout and lower data quality [50, 51]. Permission to use C-SSRS scales was obtained from the copyright holders. Ethics approval was obtained through the first author’s host university ethics committees (S1, 2017001069, H0016220; S2, H19153; S3, H20149), and studies were in accord with the World Medical Association’s Declaration of Helsinki [52]. Participants indicated consent by clicking on an ‘agree to participate’ button after reading a study information statement. With ethics committee approval, parental consent was not required for Study 1 (S1) participants aged 13+ years or S2 participants aged 14+ years, but was required for S3 participants aged 14–17. Adolescents were a target group for these studies to help validate the SS across a broad age span. Our open science approach includes youth participation rights [53, 54]. However, no student research-credit participants were included, or incentives offered, due to data validity concerns [55, 56]. Analyses were conducted with the open-source statistical environment R, v.4.1.1, *Kick Things* [57]. R code and data are available at: https://osf.io/vjxnq/.

### Procedure

S1 included the selection of suicide pool items and scales, review and revisions of items, data collection, and psychometric analyses. A multidisciplinary panel (*N* = 12) selected pool items, reviewed candidate items, evaluated linguistic and cultural validity, conducted the studies and evaluated results. Panelists came from several countries, backgrounds, and disciplines such as psychology, medicine, education, and genetics. After determining an item pool, we piloted test items with community members, asking for feedback on clarity and content. Results led to several wording changes. Next, identical online surveys were conducted for S1 in English (*N* = 5,115) and Chinese (*N* = 2,988). Description and findings of the Chinese language study are extensive and presented elsewhere [58]. S2 (*N* = 814) included SS modifications and a time-two (T2) two-week follow-up (*n* = 190). S3 (*N* = 626) tested additional revisions.

Three sequential cross-sectional surveys obtained participants through social media advertisements (e.g., Instagram, Facebook) and snowballing. Surveys were promoted approximately 2–14 weeks. Researcher-funded advertising totaled < US$3,000. Participants first read information statements, then indicated their consent to answer questions on suicide and other topics. We utilized an anonymous online platform, as anonymity can improve response accuracy on stigmatized topics [59–62]. However, to obtain T2 participants, we requested email addresses to send a survey link to, which were deleted after two invitations. Surveys included open comments and provided contacts to freely available international support services and took about 10–15 minutes to complete. To obtain a sample representing the full suicidality spectrum, S1 was promoted as a study on suicide, a method that has resulted in high participation rates by suicidal people [e.g., 63]. We used progress bars and a simple but attractive format to improve response rates [64]. Pool items were randomized to limit order bias, with the exception of items within depression scales. Demographic items were presented last to limit social desirability bias.

### Measures and factor analysis

Surveys included measures of psychopathology and positive factors. We expected the SS to show strong positive correlations with psychopathology/risk factors and negative associations with protective factors. Unless otherwise indicated, we used discrete visual analogue responses (e.g., 1 = very unlikely, 2, 3, 4, 5 = very likely), as typical Likert-type responses may be less likely to show equivalent response steps [65].

All scales were examined for factor structure, unidimensional model fit, and internal consistency (Table 1). We conducted minimum residual FA (direct oblimin rotation) with the psych package, utilizing a mixed tetrachoric and polychoric correlation matrix when accommodating dichotomous and ordered-categorical items [66]. This method provides an unweighted least squares solution, which is more robust to skewed distributions [67]. Comrey and Lee [68] considered factor loadings ≥ .71 (sharing > 50% common variance) as ‘excellent.’ Similarly, communalities (*h*^*2*^*)* ≥ .60 indicate a strong representation of the factor structure [69]. In addition, the Tucker Lewis Index of factorability (TLI) and root mean square error of approximation (RMSEA) are provided as indicators of model fit. High model fit (e.g., TLI) should be near 1.0, while error should be close to 0, but we do not apply cutoff score interpretations [70, 71]. We used the coefficientalpha package [72] to calculate robust ω, with bootstrapped 95% CI’s, as a recommended estimate of internal consistency for congeneric scales [73, 74].

**Table 1:** Factor analyses and internal consistencies of study measures.

	Minimum residual factor analysis					ω
Study/scale	TLI	95% CI	*V*	Loading	*h* ^*2*^	95% CI
Study 1 (*N* = 5115)						
SWLS	.98	.09	.65	.68 - .90	.46 - .81	[.88, .89]
PHQ-9	.89	.12	.56	.65 - .86	.42 - .75	[.89, .90]
PHQ-8	.91	.12	.55	.66 - .83	.44 - .69	[.87, .88]
DASS-Anxiety	.96	.09	.63	.63 - .88	.40 - .77	[.89, .90]
DASS-Depression	.94	.14	.74	.81 - .88	.65 - .78	[.93, .93]
C-SSRS-10	.82	.21	.70	.64 - .96	.41 - .92	[.87, .88]
C-SSRS-5	.98	.06	.54	.56 - .83	.31 - .69	[.85, .86]
SABCS-m	.87	.20	.65	.54 - .87	.29 - .76	[.91, .92]
Study 2 (*N* = 814)						
MSPSS Family	.98	.12	.81	.86 - .94	.77 - .91	[.94, .95]
MSPSS Friends	.97	.14	.81	.88 - .93	.78 - .86	[.94, .95]
DASS-Anxiety	.98	.06	.60	.52 - .86	.27 - .74	[.90, .91]
DASS-Depression	.97	.10	.71	.75 - .88	.56 - .77	[.94, .95]
SABCS-m	.93	.16	.71	.69 - .91	.48 - .83	[.93, .94]
Study 3 (*N* = 626)						
PROMIS ES-m	.97	.14	.86	.88 - .95	.78 - .90	[.96, .96]
PROMIS-D-m	.97	.11	.84	.89 - .95	.80 - .90	[.96, .97]
PROMIS-A-m	.97	.10	.72	.78 - .92	.61 - .84	[.95, .95]
SABCS-m	.96	.11	.67	.54 - .90	.29 - .81	[.93, .94]

*Note*. TLI = Tucker-Lewis Index; RMSEA = root mean square error of approximation; *V* = common variance; *h*^*2*^ = communalities; ω = internal consistency, 95% CI (bootstrapped 1000 iterations); SWLS = Satisfaction with Life Scale; PHQ = Patient Health Questionnaire; DASS = Depression Anxiety Stress Scales; C-SSRS-10 = Columbia–Suicide Severity Rating Scales, 10-item screener; C-SSRS-5 = 5-item ideation scale; SABCS = Suicidal Affect-Behavior-Cognition Scale; MSPSS = Multidimensional Scale of Perceived Social Support; PROMIS = Patient-Reported Outcomes Measurement Information System scales, ES = emotional support, D = depression, A = anxiety; m = modified.

S1 included the Satisfaction With Life Scale [75], a five-item measure of global satisfaction with life. The Patient Health Questionnaire-8/9 [76, 77] assessed participants’ somatic and non-somatic depressive symptoms and contributed one pool item (Dead). The Depression Anxiety Stress Scales [78] included two seven-item scales assessing past-week non-somatic symptoms of depression, and somatic and non-somatic anxiety symptoms (DASS-A), on four-point response sets. The depression scale contributed one pool item (Meaning).

S2 included the Multidimensional Scale of Perceived Social Support’s [79] two four-item subscales assessing perceived social support from family and friends. S3 included three freely available Patient-Reported Outcomes Measurement Information System^®^ scales [45]. We modified PROMIS^®^ scales by converting Likert-type to discrete visual analogue responses. Emotional Support v2.0–6a [80] is a six-item measure of current feelings of being emotionally supported and valued. The Emotional Distress Depression Scale v1.0–8a [81] consisted of eight items measuring past-week non-somatic depressive symptoms. The PROMIS-A v1.0–8a [81] measured participants’ non-somatic anxiety symptoms.

#### SRAs

All studies included the SABCS [27]. Legacy SABCS includes six items assessing affective, behavioral, and cognitive suicidal attributes. As recommended [40], the behaviors item was expanded to three items: Ideation-lifetime, Plan-intent, and Attempt-intent, which were graded by intent to die. Panel discussions and community feedback led to the following modifications. Wish to live (WTL) and wish to die (WTD) timeframes were changed from ‘right now’ to ‘recently’ to include a longer but contemporary affective state. Similarly, Debate was changed from ‘ever’ to ‘in the past year.’ We calculated a modified version (SABCS-m) for scale comparisons, which included: Debate, Ideation-year, WTD, WTL (reverse-scored), Predict (prediction of future suicide attempts), and Attempts-intent.

Two C-SSRS scales [37] were included for the item pool and scale comparisons. The C-SSRS self-report screener (C-SSRS-10) includes ten yes/no items, and has received several notable endorsements [e.g., 82]. Ideation-binary has been used as a gateway item. Those responding ‘yes’ complete all items, those responding ‘no’ only complete Sleep and Plan-binary items. We asked participants to complete all items. The five-item suicidal ideation intensity scale (C-SSRS-5) is scored on six-point Guttman response sets, differing for each item. Wording was taken directly from the clinical scale with minimal modifications for self-report.

#### Pool items & selection (S1)

Over 200 items from over 50 SRAs were reviewed for inclusion. Most instruments overlapped with identical or similar items on cognition (suicidal thoughts), behaviors (suicide plans and attempts), and less often–affect (desire to live/die). In contrast to most SRA studies, we included items on the internal suicidal debate (Debate, RFD). There were many minor wording differences, often on timeframe (e.g., past 7 days, lifetime), synonyms (e.g., kill yourself, end your life), and response options. Many used dichotomous responses, some used ordered behavioral frequencies (e.g., once a week, 2–5 times a week). Most used Likert or Guttman-type ordered-categorical responses. We considered psychometric properties and popularity, aiming for diversity among validated suicidal facets. Item selection was informed by theory, such as Shneidman’s [16] commonalities of suicide and the suicidal barometer model [27].

We included three popular single-item (SI) SRAs: the PHQ-9’s Dead; the BDI-II’s Ideation-BDI; and the Hamilton Depression Rating Scale’s [83] Wish-HAMD, which is similar to the Quick Inventory of Depressive Symptomatology SI [84]. These single-item SRAs have been used in numerous studies and clinical settings [e.g., 85–87]. It is also noteworthy that several items owe their roots to the SSI and other early instruments but have been modified. In addition to wording changes, we sometimes expanded response sets as evidence shows 4–7 points are usually ideal [88, 89]. Ultimately, S1 included 30 pool items (Appendix A in S1 File).

### Analyses

Analyses followed expert advice which proposes that multiple fit indices are useful for improving measurement models, but that cutoffs should not be used to accept or reject models [e.g., 9, 70, 90]. We did not use CFA due to unmet tau equivalence assumptions, and as FA and BA are more suitable for identifying the true underlying structure [23, 91]. Rest-score plots examined monotonicity and linearity [45, 92]. We used the psych package [66] for hierarchical cluster analysis (CA), FA, and BA. IRT analyses used the ltm package [93].

CA indicates the ideal number of clusters and item loadings. It includes an estimate of model fit and error (root mean square residuals, RMSR). In addition, CA analyses provide a graphic illustrating cluster hierarchies to examine item associations.

We conducted BA using Schmid-Leiman oblique rotations [94]. BA includes general factor item loadings and communalities comparable to FA, and additional common variance unique to item grouping factors [66, 95]. In addition, BA provides explained common variance (ECV), an indicator of unidimensionality, McDonald’s ω_h_ as an estimate of common latent trait variance, and model error (RMSEA) [73, 74]. We examined both general and group factors, however, for scale diagnostics, we focus on general factor statistics as our aim was to identify core latent trait attributes. Group factor trends are presented for discussion.

With IRT, the latent trait is quantified as theta, with scores typically ranging from -4.0 to 4.0. Higher values indicate higher trait levels. Analyses provide item discrimination/slope (*a*) and information functions (IF), which inform us of the item’s ability to discriminate individuals on latent trait levels, and how much information they provide, respectively. IRT also provides item response category cutpoints (*b*), and graphics illustrating IF and *b* values, helping to identify problems in monotonicity, number of item responses, uninformative items, and total test information. We determined the graded response model [GRM; 96] fit data best as it allows for variance in item discrimination and response formats, if responses are graded (increasing/decreasing or dichotomous).

We also calculated empirical Bayesian estimates of individual ability estimates. Ability scores are GRM-derived theta values based on individual item characteristics, and unique scale response patterns. These were compared with traditional sum scores.

We assessed test-retest relative reliability through Pearson’s *r*, and absolute reliability via intraclass correlation coefficient (ICC_3,1_; two-way mixed model, absolute agreement, single measure). Younger ages (e.g., aged 13 only, 13–15) were examined for unique response patterns through item/scale diagnostics [97]. We also checked item and test invariance by demographics (e.g., age, first language) and clinical factors (self-reported psychiatric diagnosis, lifetime suicide attempts) through differential item functioning (DIF) and differential test functioning. This approach has demonstrated superiority over CFA and other invariance tests [98]. We used the lordif package [99], which conducts an iterative hybrid ordinal logistic regression based on GRM modeling to detect DIF through *R*^*2*^ change (≥ .02), which is preferable to using sum scores and is robust to non-normally distributed data.

### Data treatment

Data cleansing involved identification and treatment of missing values, univariate and multivariate outliers, and inauthentic responses [100–103]. We considered missingness, Mahalanobis’ distance scores, and used the careless package [104] to identify psychometric antonyms and long strings, to guide removal on a case-by-case basis. Item pool missing values totaled 7.5%, S2 missing = 5.2%, S3 = 10.4%. Missing values were replaced through expectation-maximization, a recommended single-input method [105, 106]. Gender and ethnicity were dichotomized (male/female sex, Euro-Caucasian/other) for some analyses. We conducted bootstrapping (1,000 iterations) to better approximate population statistics and correct for deviations from normal distributions [107, 108].

## Results

### Participants

S1 participants (*N* = 5,115) were aged 13–82 years (*M* = 18.64, *SD* = 7.98); 58.3% female, 37.1% male, 4.6% nonbinary^+^; 56.8% identified as Euro-Caucasian, 43.2% as various other ethnicities; 68.8% were native English speakers; 13.2% were from Australia or New Zealand, 53.4% from the UK, 19.0% from Southeast Asia, 7.2% from Canada or the USA; 19.5% were from urban areas, 62.5% from suburbs/towns, 18.0% from rural/remote areas; 33.3% self-reported a history of psychiatric diagnosis; 36.9% indicated no financial distress, 3.9% indicated high distress; 57.7% had not (yet) completed high school, 1.2% held postgraduate degrees.

S2 included 814 participants, aged 14–80 years (*M* = 25.60, *SD* = 12.05); 58.7% female, 35.4% male, 5.9% nonbinary^+^; 67.9% identified as Euro-Caucasian, 10.2% Asian, and 21.9% as various ethnicities; 35.5% were from Australia or New Zealand, 16.5% from the UK, 7.2% from the US or Canada, 8.8% from Asian countries, and 31.7% from several others; 28.7% were from urban areas, 61.1% from suburbs/towns, 10.2% from rural/remote areas; 33.8% had not (yet) completed high school, 5.3% held postgraduate degrees. For time-two (T2, two-weeks, *n* = 190), there were no statistically significant differences on demographics with T1 participants, *p*s > .05.

S3 included 626 participants aged 14–83 years (*M* = 35.85, *SD* = 17.84); 63.1% female, 31.0% male, 5.9% nonbinary^+^; 82.6% identified as Euro-Caucasian, 17.4% as various other ethnicities; 44.0% were from Australia or New Zealand, 22.5% from South Africa, 16.3% from Canada or the USA, 8.3% from the UK; 47.9% were from urban areas, 42.7% from suburbs/towns, 9.4% from rural/remote areas; 19.7% had not (yet) completed high school, 15.3% held postgraduate degrees.

Scale development requires large samples covering the full spectrum of the construct, providing sufficient data on a broad range of theta. For S1, there were 170.5 cases per pool item. All 127 response options had ≥ 86 endorsements. For the final SS, all possible sum scores had ≥ 85 cases. For S2, there were ≥ 39 cases/response option, for T2, ≥ 3 cases/response option. For S3, ≥ 17 cases/response option.

### Item selection

Analyses began by testing for unidimensionality with the item pool [109], followed by reducing items to a parsimonious set maximizing latent trait information [110]. CA showed pool items reasonably formed a single but complex cluster, fit = .96, RMSR = .07. FA results indicated a single factor explaining 66% common variance, TLI = .47, RMSEA = .27. BA results were more ambiguous, showing a moderately strong general factor, and three weak to moderate group factors, ω_h_ = .83. We compared unconstrained GRM (items may vary in discrimination/slope levels) vs. constrained (items discriminate equally on theta). ANOVA results showed the unconstrained model fit best, with less information loss, ΔAIC = 5,013, *p* < .001. We therefore conducted unconstrained GRM. Table 2 shows pool diagnostics, revealing weak to very strong items.

**Table 2:** Item pool cluster, factor analysis, bifactor analysis and graded response modeling.

Item	Clus	FA		BA		GRM		
		L	*h* ^*2*^	g	*h* ^*2*^	b_l_	b_u_	*a*
Dead	.80	.84	.71	.76	.71	-0.82	0.92	2.40
Ideation-year	.83	.87	.76	.78	.73	-1.50	0.69	2.73
Debate	.85	.89	.79	.79	.73	-1.22	0.87	2.81
Predict	.84	.88	.77	.78	.73	-0.85	1.56	2.71
Desire to kill self	.88	.91	.83	.83	.87	-0.75	1.98	3.74
Meaning	.76	.79	.63	.72	.69	-1.17	0.96	2.08
Wish to die	.85	.86	.74	.81	.85	-1.11	1.92	2.78
Reasons for dying	.78	.82	.66	.74	.72	-0.65	2.27	2.32
Ideation-lifetime	.82	.91	.83	.74	.76	-2.11	0.19	2.39
Plan-intent	.82	.87	.76	.72	.79	-1.00	1.23	2.08
Attempt-intent	.67	.76	.58	.57	.75	0.33	1.68	1.43
Wish to live-r	.76	.76	.58	.72	.71	-1.41	2.50	1.85
Ideation-times	.84	.88	.77	.79	.74	-1.40	1.94	2.86
Ideation-hours	.78	.83	.69	.72	.62	-2.17	2.09	2.14
Ideation-control	.74	.76	.58	.69	.56	-1.60	2.09	1.82
Deterrents	.55	.62	.38	.50	.30	-1.55	3.64	1.20
Reasons	.65	.68	.46	.60	.52	-1.95	1.05	1.22
Wish-HamD	.65	.69	.48	.60	.43	-1.12	2.42	1.37
Ideation-BDI	.76	.94	.88	.72	.65	-0.74	1.95	2.51
Save	.76	.80	.64	.71	.64	-0.60	1.96	2.13
Sleep-b	.56	.84	.71	.52	.43	-1.52	--	1.92
Ideation-b	.62	.94	.88	.58	.60	-1.34	--	2.47
How-b	.66	.86	.74	.61	.51	-0.78	--	2.00
Intent-b	.75	.90	.82	.67	.62	-0.03	--	2.26
Plan-b	.59	.81	.65	.53	.44	1.13	--	2.17
Attempt-b	.62	.77	.60	.52	.69	1.00	--	1.66
Self-harm-b	.50	.65	.43	.46	.30	-1.16	--	1.08
Stopped-b	.58	.73	.53	.50	.52	0.84	--	1.31
Stop-self-b	.58	.71	.50	.50	.44	0.41	--	1.15
Prepare-b	.68	.82	.68	.60	.56	0.43	--	1.62

*Note*. *N* = 5,115. Clus = hierarchical cluster analysis, FA = minimum residual factor analysis, BA = bifactor analysis, GRM = graded response model, L = loading, g = general factor, *h*^*2*^ = communalities, b_l_ = lower threshold, b_u_ = upper, *a* = discrimination, r = reverse scored, b = binary.

We repeated analyses, removing the worst fitting item one by one. When an item is removed, theta is redefined by remaining items as we refine the model. Behavior items (self-harm, attempts, plans) were among the weakest and were removed early. We found 16 items with strong psychometrics. Analyses were repeated with subsamples (e.g., aged 13 [*n* = 355], aged 13–15 [*n* = 1917], aged 40+ [*n* = 224], native English vs. non). Some items, such as Plan-ever, Ideation-lifetime and Ideation-control, were removed due to weaknesses with multiple groups. An 11-item set included four items that were valuable but with shortcomings: Ideation-BDI, Save, Ideation-times, and Meaning. Ideation-BDI showed weaknesses with youth and monotonicity. Save showed comparatively lower performance overall and with youth, and some linearity issues with non-native English speakers and non-Euro-Caucasians, so was removed. We compared two similar items: Ideation-year and Ideation-times. They differ by the subjective ‘often’ vs. specific frequency (e.g., 2–5 times a week). Ideation-times showed comparative weakness with older participants, while Ideation-year showed slightly higher discrimination for the full sample (2.84 vs. 2.63) and was therefore selected as the stronger item. We retained Meaning as it performed well overall, only showing slightly lower properties with more extreme age groups. We also thought it might benefit by rewording and expanding response points.

### Suicidality Scale psychometrics

**Fig 1:**
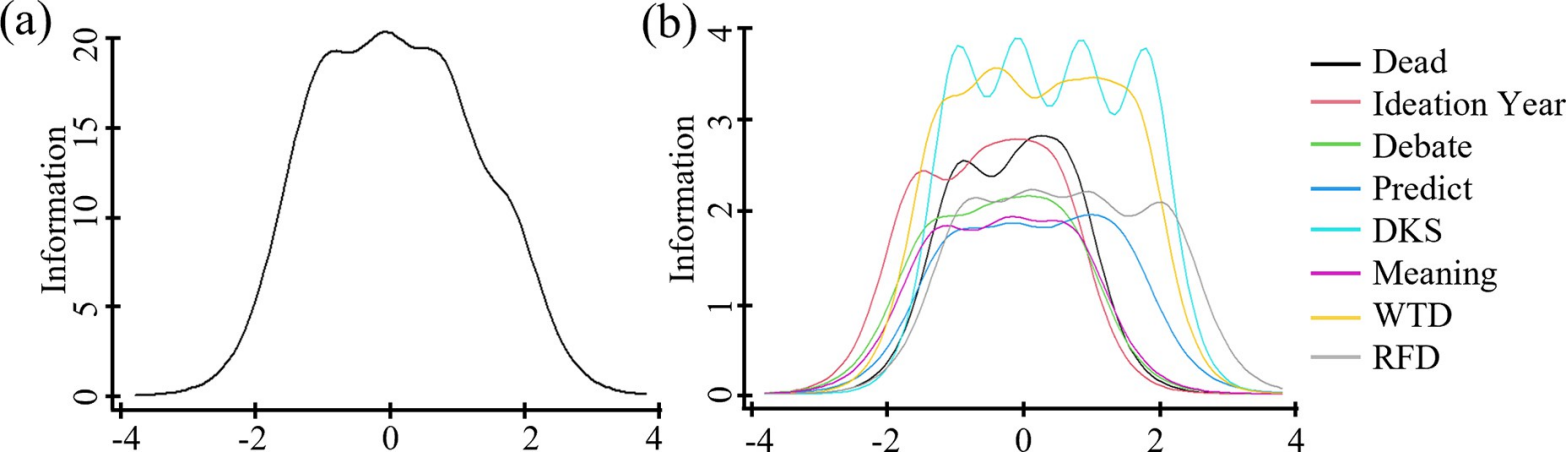
Suicidality Scale test information (left) and item information curves (right).

We found eight items provided a highly informative measure across the full sample and subsamples–forming the Suicidality Scale. Table 3 shows high but variable item discrimination and information functions, supporting decisions to treat items as non-uniform indicators of suicidal attributes. We also see important variations in item abilities to discriminate at the lowest and highest trait levels. Ideation and Debate captured more information at low levels, while RFD and DKS did so at high suicidality levels. Fig 1 illustrates the GRM output in Table 3. The breadth of item thresholds (*b* values) indicates theta coverage. The volume under each item’s line indicates the amount of information captured on the latent trait.

**Table 3:** Suicidality Scale item statistics (sample 1, *N* = 5,115).

Item	Clus	FA		BA		Graded response model			
		L	*h* ^*2*^	G	*h* ^*2*^	*b*_*l*_	*b*_*u*_	*a*	IF
DKS	.92	.93	.86	.93	.89	-0.88	1.84	3.79	13.19
WTD	.91	.91	.83	.92	.85	-1.20	1.61	3.29	12.33
RFD	.83	.83	.68	.85	.73	-0.89	1.98	2.64	8.15
Ideation-year	.84	.84	.70	.79	.83	-1.55	0.44	2.81	7.46
Dead	.85	.85	.72	.83	.75	-0.96	0.61	3.01	6.93
Predict	.83	.83	.69	.83	.72	-0.99	1.33	2.40	6.39
Debate	.85	.85	.72	.81	.80	-1.26	0.60	2.57	6.34
Meaning	.82	.82	.67	.83	.73	-1.19	0.66	2.36	5.33

*Note*. *b*_*l*_ = lower item threshold, *b*_*u*_ = upper, *a* = discrimination, IF = information function, Clus = hierarchical cluster loading, FA = minimum residual factor analysis, BA = bifactor analysis, L = common factor loading, *h*^*2*^ = communality, g = general factor.

**Fig 2:**
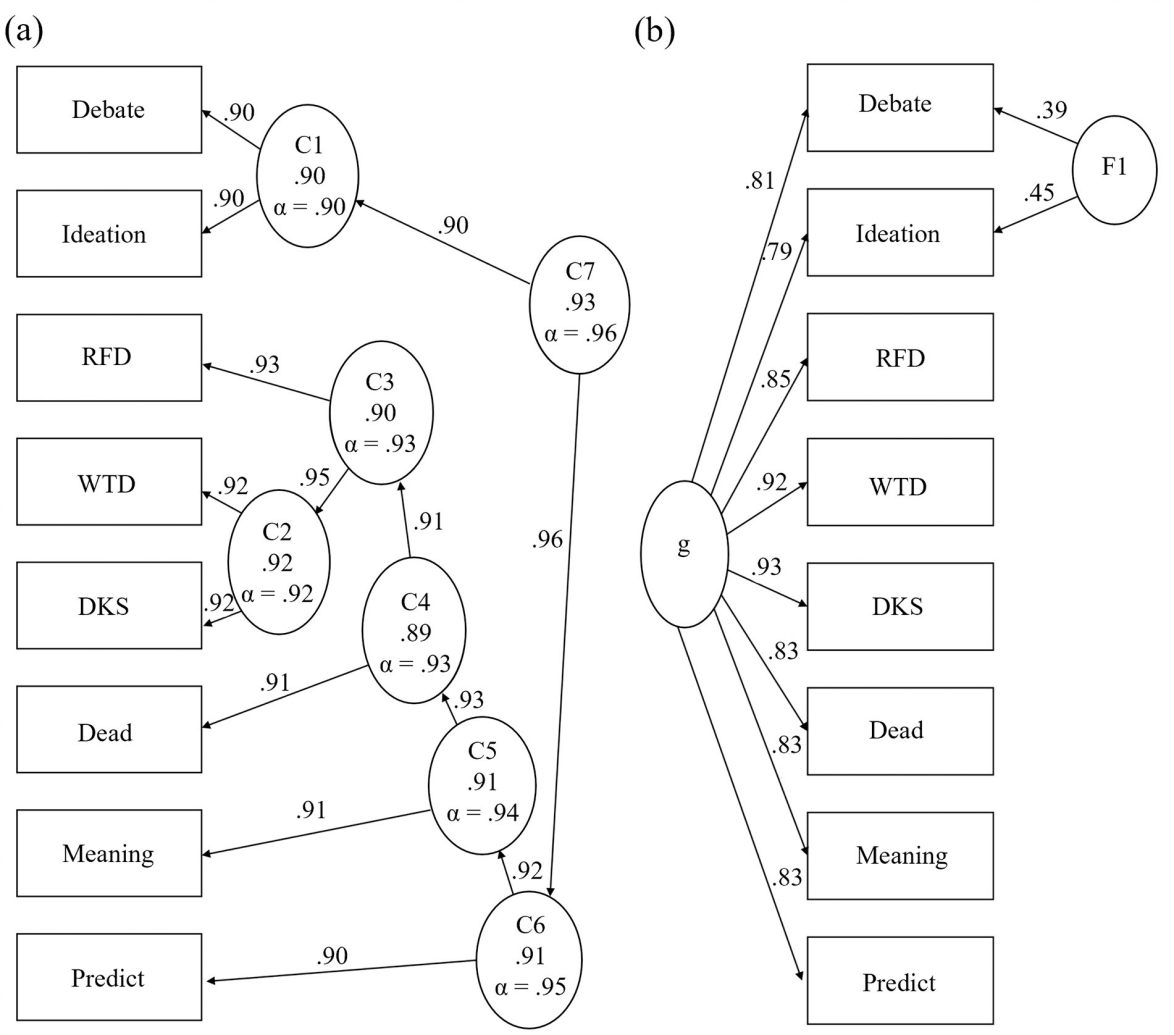
Hierarchical cluster analysis (left) and bifactor analysis (right) of the Suicidality Scale, (S1, N = 5,115). Ellipses represent assumed latent traits, rectangles represent observed traits (item responses), g = general factor, F = group factor.

Fig 2 shows the SS hierarchical cluster pathways and BA group and general factor associations. Note that the algorithm attempts to determine three meaningful group factors [109]. However, only one group factor with loadings ≥ .20 was identified in S1.

**Fig 3:**
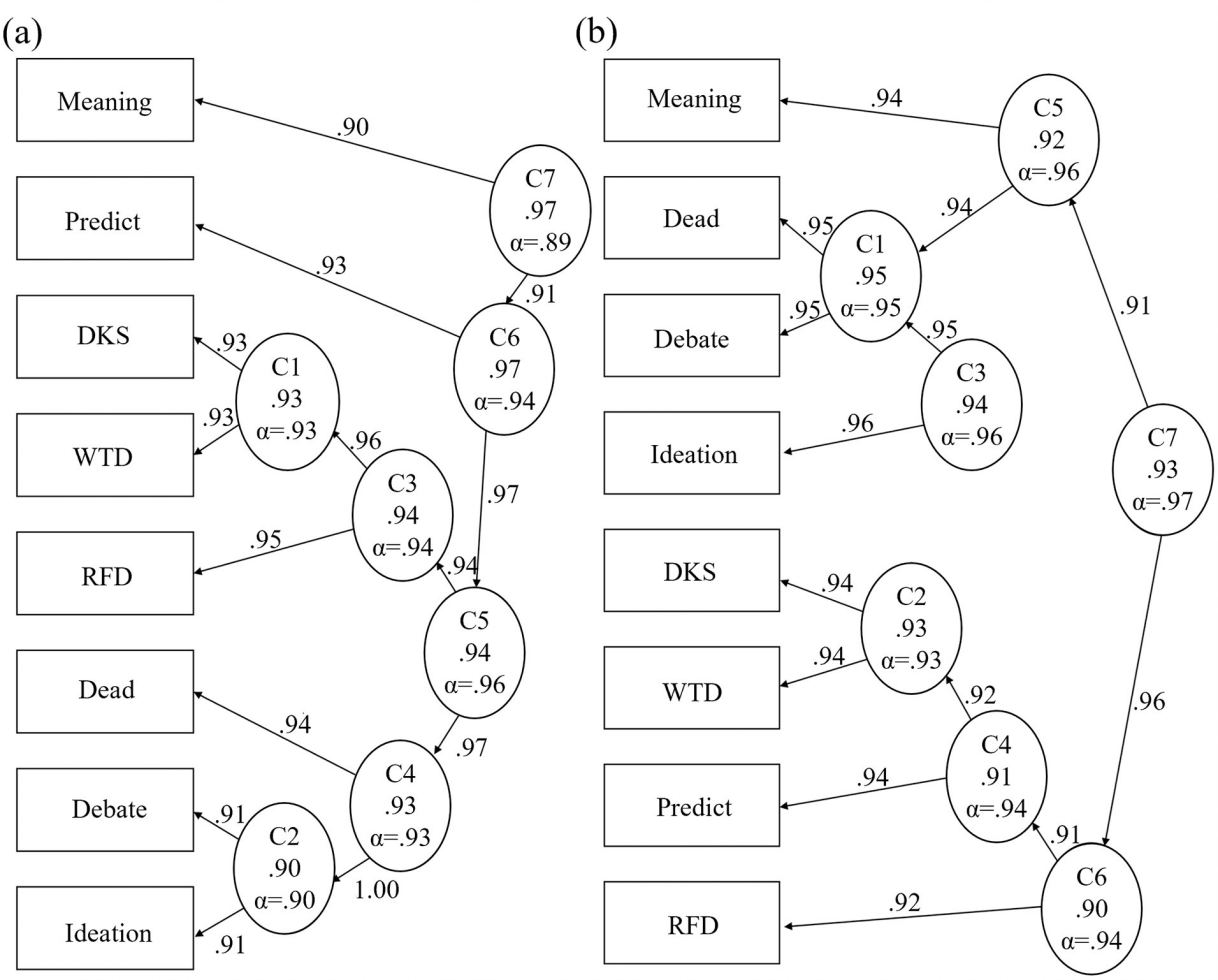
Suicidality Scale hierarchical cluster analyses, studies 2 (left) and 3 (right). Ellipses represent assumed latent traits, rectangles represent observed traits (item responses). These results show modest trends of item clusters that graded response model analyses showed strengths at low theta levels (Debate, Ideation, Dead, Meaning), and at high theta levels (RFD, WTD, DKS, Predict).

**Fig 4:**
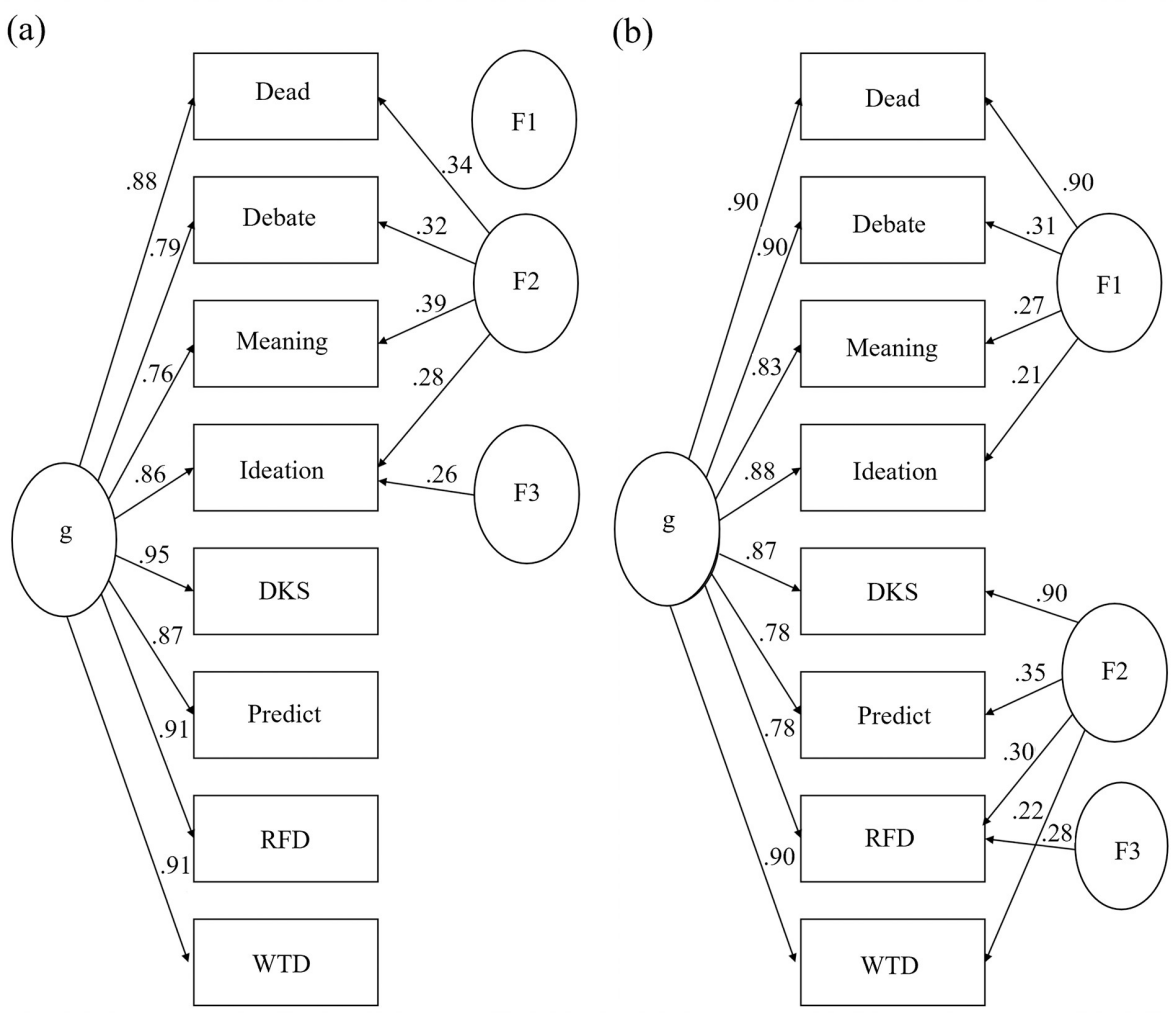
Suicidality Scale bifactor analyses, studies 2 (left) and 3 (right). Ellipses represent assumed latent traits, rectangles represent observed traits (item responses), g = general factor, F = group factor. These results show limited evidence of two groups, one that graded response model analyses showed strengths at low theta levels (Debate, Ideation, Dead, Meaning), and one at high theta levels (RFD, WTD, DKS, Predict).

Figs 3 and 4 show S2–S3 cluster and BA diagrams, respectively. Item group associations may help us understand the nature of the latent trait. Across the three studies, we see weak to moderate evidence of two subgroups, more evident in S3.

**Fig 5:**
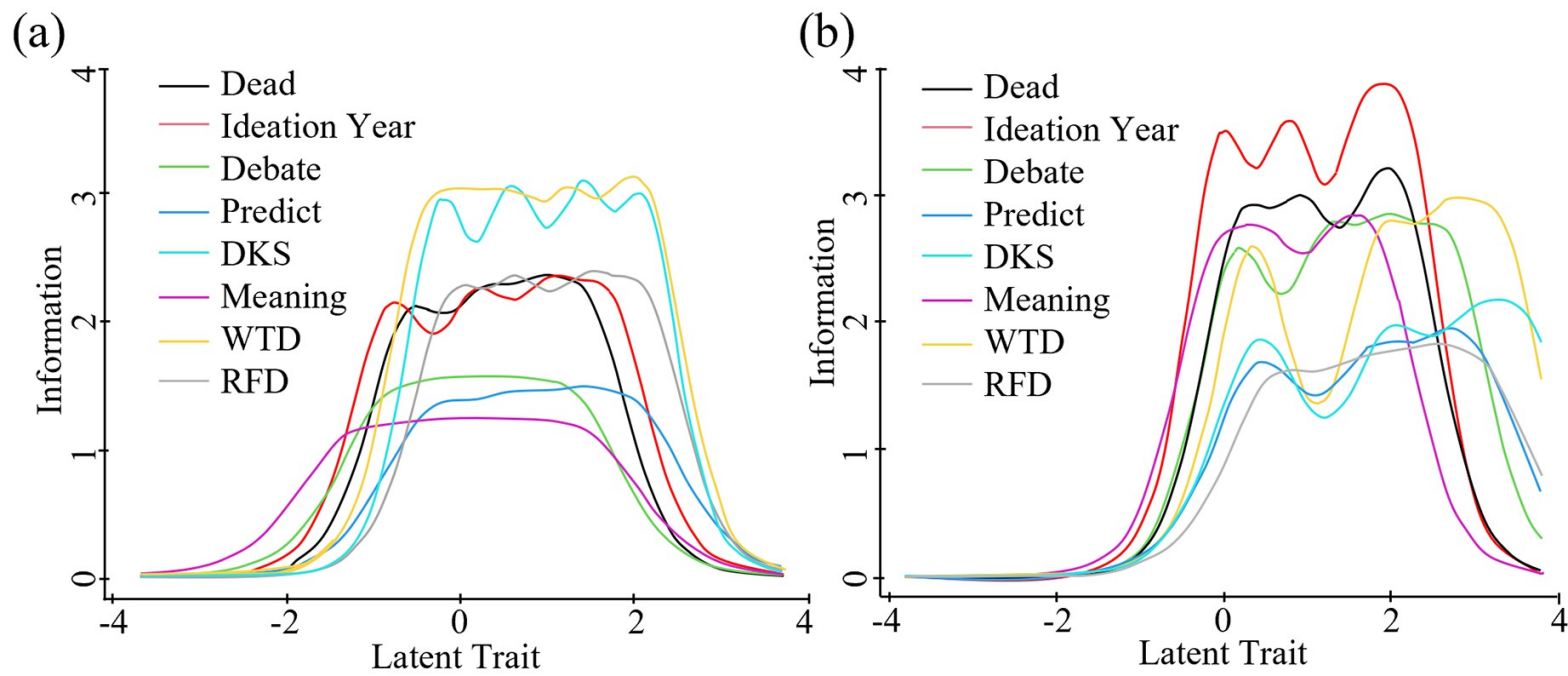
Suicidality Scale item information curves for study 2 (left) and study 3 (right).

Fig 5 illustrates S2 and S3 results. In S3, we see the upper end of theta is not well defined, likely due to the smaller sample size and fewer participants at higher suicidality levels. Table 4 presents SS model fit statistics for all studies, demonstrating a strong, if imperfect, measure with high fit, low error, and high internal consistency.

**Table 4:** Suicidality Scale model diagnostics.

	Cluster		FA		BA				
Study	Fit	RMSR	RMSEA	*V*	ω_h_	ECV	RMSEA	ω	95% CI
S1	.98	.03	.12	.74	.94	.92	.05	.96	[.96, .96]
S2	.99	.03	.14	.84	.93	.91	.06	.97	[.96, .97]
S3	.99	.04	.24	.87	.93	.87	.02	.97	[.96, .97]

*Note*. S1, *N* = 5115; S2, *N* = 814; S3, *N* = 626; FA = minimum residual factor analysis; BA = bifactor analysis; RMSR = root mean square of residuals; TLI = Tucker-Lewis Index of factoring reliability; RMSEA = root mean square error of approximation; ω_h_ = general factor variance; ECV = explained common variance; ω = internal consistency, bootstrapped 1000 iterations.

### Differential item & test functioning

We next performed DIF and DTF checks to determine if item or test scores differ by group membership, resulting in biased assessment. Grouping variables include 2–3 categories: age (A = 13–18, 19+; B = 13–15, 16–19, 20+; C = 13–39, 40+); gender (A = male/female/non-binary^+^; B = male/female); region = urban/town/rural; ethnicity = Euro-Caucasian/other; first language = English/other; psychiatric diagnosis yes/no; suicide attempts yes/no. No SS items, or test total, showed DF for any grouping (Δ*R*^*2*^ < .02). When examining the best 11 items (including Save, Ideation-BDI, Ideation-times) we found some evidence of DTF by age and psychiatric diagnosis, indicating that including one or more of those items results in discrepant inter-group evaluations. The lack of DIF or DTF for participants with or without a lifetime suicide attempt informs us that there was no meaningful difference in trait assessment due to attempt status. S2 and S3 DIF checks revealed no evidence of invariance by age groups, ethnicity, gender, urban/rural residence, or between South Africans (S3; *n* = 141) and others.

### Predictive ability, test-retest reliability

In S2, T2 (two-weeks) examined temporal stability of the SS and evidence of short-term predictive ability. An ANCOVA (controlling for sex, age, ethnicity) comparing participants who completed T2 (*n* = 190), with those who did not, showed no statistically significant group difference with SS T1 ability scores, *F*(1, 809) = 0.39, *p* = .53, η^2^p = .00. Partial correlations (controlling for demographics) compared T1 with T2 ability scores (derived from T2 data only), showing high temporal stability, *r* = .87, 95% CI [.81, .92] (Table 5). Measurement agreement between the two ability scores was good, ICC_3,1_ = .89, 95% CI [.^85^, .^92^].

**Table 5:** Suicidality Scale time 1 and 2 (two weeks) central tendency and partial correlations.

	T1		T2			
Item	*M*	*SD*	*M*	*SD*	*r*	95% CI
DKS	2.04	1.21	1.89	1.18	.72	[.62, .81]
WTD	2.73	1.81	2.69	1.75	.77	[.68, .85]
Dead	2.44	1.42	2.28	1.45	.78	[.70, .84]
Debate	2.68	1.46	2.64	1.47	.74	[.66, .81]
Ideation	2.51	1.37	2.44	1.39	.82	[.74, .87]
Predict	2.09	1.26	2.00	1.27	.78	[.70, .85]
Meaning	2.83	1.39	2.67	1.37	.71	[.62, .77]
RFD	1.97	1.19	1.89	2.10	.74	[.65, .83]
SS sum	19.29	9.99	18.51	9.72	.87	[.82, .92]
SS ability	-0.05	1.16	0.08	1.11	.87	[.82, .92]

*Note*. Study 2, T2 *n* = 190; sum = sum scores; ability = ability scores. Correlations control for sex, age and ethnicity (Euro-Caucasian/other), bootstrapped 1,000 iterations.

### SS revisions

Sustainable scale development includes testing modifications with the aim of making incremental improvements when warranted (see Appendix B in S1 File for revisions, Appendix C in S1 File for final SS). S1 included legacy PHQ-9 and DASS-D items that met criteria for inclusion in the SS but showed weaknesses. For S2, we increased responses from four to five, removed non-anchor labels, and reworded for clarity and consistency. Notably, we revised Dead to remove the double-barreled format. We kept ‘better off dead’ and deleted ‘hurting yourself.’ ‘Better off dead’ is more directly relevant to suicidality, and evidence shows self-harming is a separate factor from suicidality [e.g., 111]. Also, our analyses showed Self-harm was the least valid pool item. For Meaning, we added ‘your’ to make the statement ‘life is meaningless’ more personal, as suicidality is most relevant to the self [e.g., 16, 112]. For Debate, we used past year for S1 and lifetime for S2. Given slightly lower psychometric properties in S2, lifetime may be too long for that item. We also used the subjective term ‘recently’ for some items, including Dead. ‘Recently’ appeared to work well, based on item statistics. Tables 6 and 7 show all items maintained strong psychometric properties across studies.

**Table 6:** Suicidality Scale cluster, factor analysis and bifactor analysis, studies 2 and 3.

	Study 2 (*N* = 814)					Study 3 (*N* = 626)				
Item	Clus	FA		BA		Clus	FA		BA	
		L	*h* ^*2*^	g	*h* ^*2*^		L	*h* ^*2*^	g	*h* ^*2*^
Dead	.92	.95	.91	.88	.89	.92	.95	.91	.90	.91
Ideation	.91	.93	.87	.86	.89	.91	.95	.91	.88	.84
Debate	.85	.89	.79	.79	.76	.92	.95	.90	.90	.92
Predict	.86	.90	.80	.87	.77	.83	.89	.79	.78	.75
DKS	.93	.96	.92	.95	.89	.92	.96	.92	.87	.95
Meaning	.81	.86	.73	.76	.76	.85	.90	.82	.83	.76
WTD	.91	.93	.86	.91	.84	.94	.97	.96	.90	.88
RFD	.88	.92	.85	.91	.84	.81	.89	.79	.78	.77

*Note*. Clus = hierarchical cluster analysis, FA = minimum residual factor analysis, BA = exploratory bifactor analysis (Schmid-Leiman), L = common factor loading, g = general factor loading.

**Table 7:** Graded response model analyses of Suicidality Scale items, studies 2 and 3.

Item	*b*_*l*_	*b*_*2*_	*b*_*3*_	*b*_*4*_	*b*_*5*_	*b*_*6*_	*a*	IF
Study 2 (*N* = 814)								
DKS	-0.28	0.58	1.39	2.15	--	--	3.67	12.22
WTD	-0.52	0.03	0.60	1.23	1.84	2.22	3.52	9.41
RFD	-0.14	0.65	1.47	2.17	—	—	3.17	11.11
Ideation	-0.97	-0.18	0.63	1.27	—	—	3.60	11.97
Dead	-0.66	0.11	0.61	1.12	—	—	3.53	10.66
Predict	-0.24	0.51	1.19	1.89	—	—	2.42	6.43
Debate	-1.32	-0.43	0.36	1.08	—	—	2.79	7.81
Meaning	-1.53	-0.43	0.49	1.29	—	—	2.34	6.92
Study 3 (*N* = 626)							
DKS	0.43	1.99	3.00	3.65	—	—	2.71	8.58
WTD	0.35	1.90	2.69	3.33	—	—	3.21	10.43
RFD	0.63	1.67	2.45	3.11	—	—	2.45	6.76
Ideation	-0.05	0.79	1.68	2.20	—	—	3.65	11.55
Dead	0.19	0.95	1.81	2.18	—	—	3.28	9.24
Predict	0.43	1.77	2.66	3.00	—	—	2.60	7.07
Debate	0.16	1.23	1.98	2.71	—	—	3.16	9.95
Meaning	-0.11	0.52	1.37	1.86	—	—	3.08	8.50

*Note*. *b* = item threshold, *a* = item discrimination, IF = information function.

**Fig 6:**
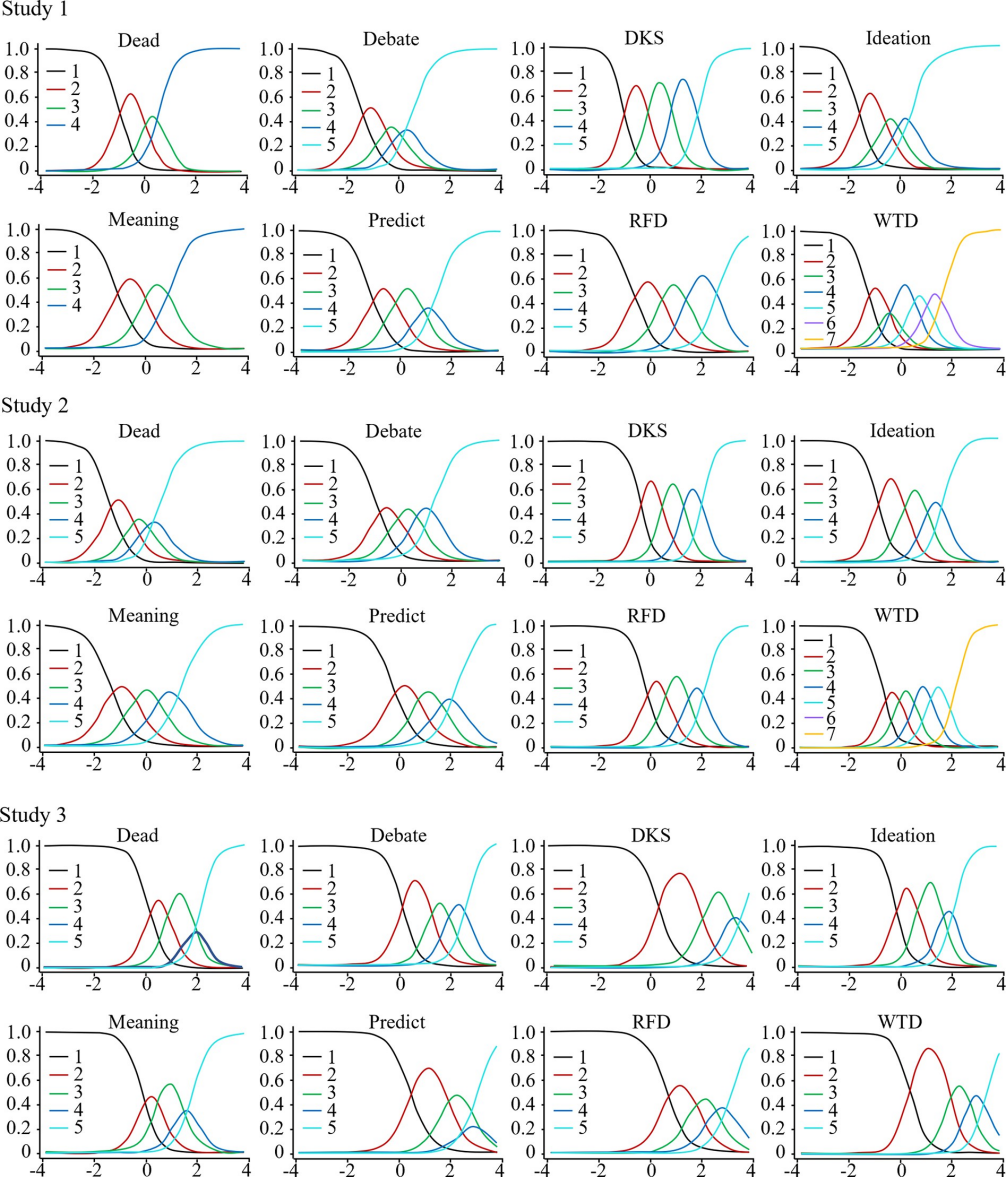
Suicidality Scale item response characteristic curves. Y-axes indicate probability of responding, x-axes indicate theta (latent trait levels).

Item response characteristic curves assist in checking monotonicity and response set validity. Fig 6 shows, for S1, all item responses were appropriately aligned on theta, with no apparent violations of monotonicity. However, for the Dead and Debate items, the second-highest options were not well-supported, indicating revised response sets or other adjustments may be helpful. In addition, WTD showed seven points may be too many as the fifth option was under-endorsed. These variations in item response characteristics, including different locations on theta (*b* values) for specific item responses, are further evidence against tau-equivalence. In S3, WTD diagnostics were strong with five response points. In S3 we also see that five points appears to be too many for Predict, however, that item captures relatively more information on high theta levels and S3 had fewer highly suicidal participants.

### Ability scores and SS associations

We next examined associations between SS ability scores and psychosocial variables, including available SRAs, controlling for demographics. Table 8 shows correlations were in expected directions, positive with psychopathology and negative with protective factors. Note that the SS shared three items with the SABCS-m, and single items with the DASS-D (Meaning) and PHQ-9 (Dead) in S1, which were later revised.

**Table 8:** Partial correlations between psychosocial variables and Suicidality Scale ability scores.

	S1 (*N* = 5,115)		S2 (*N* = 814)		S3 (*N* = 626)	
Variable	*r*	95% CI	*r*	95% CI	*r*	95% CI
PROMIS-Anxiety-m	—	—	—	—	.56	[.50, .62]
DASS-Anxiety	.64	[.60, .67]	.63	[.58, .68]	—	—
DASS-Depression*	.82	[.80, .84]	.77	[.73, .80]	—	—
PHQ-8 Depression	.76	[.73, .79]	—	—	—	—
PROMIS-Depression-m	—	—	—	—	.73	[.69, .77]
PHQ-9 SI*	.85	[.84, .87]	—	—	—	—
BDI-II SI	.77	[.75, .80]	—	—	—	—
HAM-Depression SI	.59	[.55, .64]	—	—	—	—
C-SSRS-10	.77	[.74, .80]	—	—	—	—
C-SSRS-5	.87	[.85, .89]	—	—	—	—
SABCS-m*	.98	[.97, .98]	.95	[.94, .96]	.97	[.97, .98]
Suicidality Scale sum	.99	[.99, .99]	.98	[.98, .99]	.98	[.98, .98]
SWLS	-.70	[-.73, -.67]	—	—	—	—
MSPSS Family	—	—	-.58	[-.62, -.53]	—	—
MSPSS Friend	—	—	-.37	[-.44, -.31]	—	—
Emotional support	—	—	—	—	-.53	[-.59, -.46]

*Note*. Correlations are between Suicidality Scale ability scores and scale sum scores, controlling for age, sex, ethnicity (Euro-Caucasian/other), first language (S1, English/other), PROMIS = Patient-Reported Outcomes Measurement Information System^®^, DASS = Depression Anxiety Stress Scales; PHQ = Patient Health Questionnaire; BDI-II = Beck Depression Inventory-II (Ideation-BDI), HAM = Hamilton Depression Rating Scale (Ideation-HAMD), C-SSRS-10 = Columbia Suicide Severity Rating Scale screener, C-SSRS-5 = 5-item clinical scale, SABCS = Suicidal Affect-Behavior-Cognition Scale, MSPSS = Multidimensional Scale of Perceived Social Support, SI = single item measure, m = modified. Bootstrapped 95% CIs, 1,000 iterations. All correlations, *p* < .0001.

*The SS shared three items with the SABCS-m, and single items (S1 only) with the PHQ-9 SI and DASS-D.

We next tested the question–does the measure matter? We compared correlations between industry standard C-SSRS sum scores and depression sum scores (CTT method), with SS ability and depression ability scores in S1. We avoided autocorrelation by using the PHQ-8 and the DASS-D-6 (removing Meaning), and statistically controlled for age, sex and ethnicity. Bootstrapped CTT analyses showed the C-SSRS-10 correlated with both depression measures .57 - .61.The C-SSRS-5 correlated with both between .65 - .71. SS ability scores correlated with the PHQ-8 at .76 - .78 and the DASS-D-6 at .83 - .84. Steiger’s *Z*-scores, comparing correlations between conventional and proposed measurement, showed *Z*s > 20.0, *p*s < .0001. Large effect sizes for all comparisons indicate meaningfully higher correlations with the SS and depression scores. The SS also showed a large effect size difference between correlations with the two depression measures, *Z* = 14.10, *p* < .0001.

We then tested the CTT hypothesis that higher sum scores necessarily indicate higher levels of the latent trait. Results did not support the hypothesis as we saw notable overlap in ability scores for specific sum scores. For example, an SS sum score of 21 (ability range = -0.49 –-0.08) includes those with theta lower than some cases with a sum of 18 (range = -0.97 - -0.40), and higher than some with a sum of 24 (range = -0.12–0.17).

## Discussion

This project was aimed at demonstrating sustainable scale development through validating a more precise measure of the latent trait suicidality. Through consecutive studies and revisions, the eight-item Suicidality Scale demonstrated high psychometric properties by capturing facets most relevant to the construct. Tests showed the SS performed well across several demographic groupings, and by mental disorder and suicide attempt history (self-reported yes/no). The strength of these findings across diverse samples and groups provides strong evidence that the SS measures common suicidality characteristics, fulfilling the core requirement of scale validity–it measures what it is supposed to measure.

It is notable, but not surprising, that no dichotomous items demonstrated sufficient validity for inclusion in the final scale. Behavior items also showed weaknesses compared with affective and cognitive items. These findings extend on the SABCS study [27], which used IRT and FA to determine a valid measure. Authors, however, allowed theory to rationalize retaining a moderately valid behavior item. Our findings provide further evidence that suicidal behaviors are meaningful facets of suicidality but items, in numerous variations, have not demonstrated sufficient validity for accurate risk assessment. Additionally, we found no support for including a self-harming item. That area benefits from unique construct-specific research [e.g., 113]. We also found no DIF by suicide attempt history, indicating the underlying trait can be assessed equivalently regardless of attempt status. This is also evidence against the hypothesis that attempt status alone can define risk. Nevertheless, behavior items remain important for biographic data and clinical evaluations.

### Mapping the suicidal mind

Hierarchical cluster and bifactor analyses revealed two possible four-item groups. GRM-derived item threshold statistics show one set appeared best at capturing information at the lowest assessed suicidal levels: Ideation, Meaning, Debate, and Dead. These findings indicate that early or low suicidality may be characterized by infrequent thoughts of suicide, with some thoughts revolving around an active suicidal debate. Feeling that one’s life has no meaning provides an affective element. The evidence here confirms that suicidality is more than simple behaviors or thoughts, it includes an internal struggle between choosing life or death [114–116]. Evans and Farberow [117] presented life/death ambivalence as possibly the most important aspect of the suicidal mind. With follow-up study, we may be able to verify such early, or lower-risk, phases.

We also saw some consistency in items capturing information at the highest theta levels: RFD, DKS, WTD, and Predict. RFD, like Debate, is directly related to life/death ambivalence [16] and suicidal debate theory [114]. DKS and WTD provide affective suicidal facets. It may be that focus on the finality of one’s decision, to kill self, to die, is highly relevant to suicidal people at their greatest risk of performing such behaviors [118]. ‘Predict’ provides further weight to concluding that debate with action. With precise SRAs, we may improve our understanding of how the suicidal mind develops and sometimes transitions into high-risk behaviors.

### Clinical decisions and cutoff scores

The greatest challenge for SRAs may be in translating assessments into appropriate clinical directions. Clinical decisions are often ordered, ranging from no treatment needed to emergency care. Many scales include attractive cutoff scores (e.g., SSI, C-SSRS), for low to high risk. However, those cutoffs were established through highly questionable ROC and AUC analyses. For SRAs, such cutoffs are based on three disproven hypotheses: 1) all items are equal in quantifying suicidality; 2) responses of all items are equally graded; 3) binary outcomes (e.g., suicide attempt vs. no attempt, high suicidality vs. low suicidality) are true dichotomies. Our research replicates previous studies demonstrating a lack of SRA tau equivalence through FA [15, 111] and IRT analyses [25, 27]. Therefore, the predictor variable, the SRA, cannot produce valid cutoff scores as sums include items and response steps of unequal weights and increments. As we saw here, SS sum scores of 21 can represent a range of latent trait levels.

**Fig 7:**
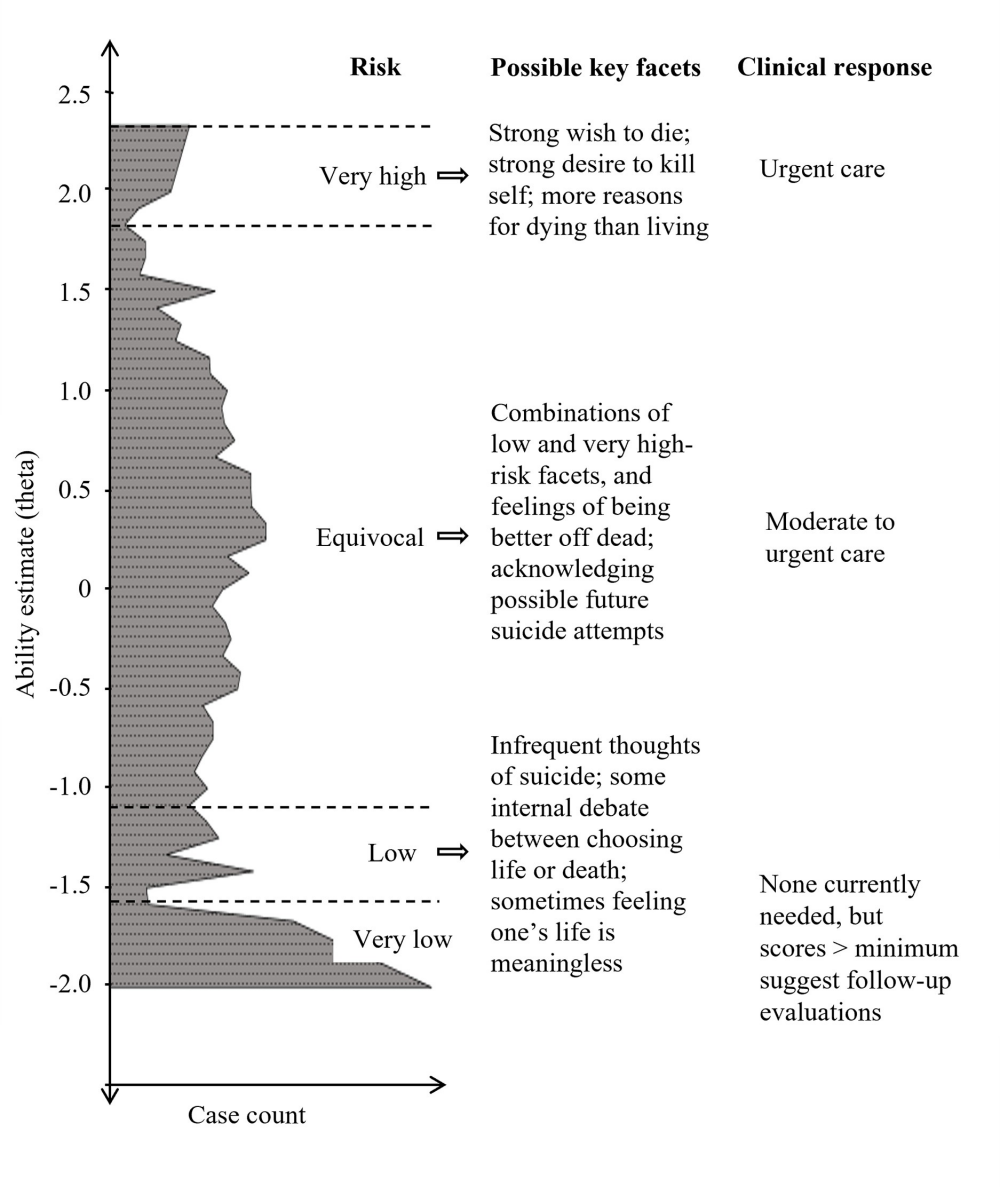
Suicidality Scale ability score frequencies, observed facets, and clinical directions. Study 1 data, *N* = 5,115. Key facets were observed across studies but require further validation. Risk levels are approximate and can change quickly. Clinical decisions require additional psychobiological data.

Given the lower validity of sum scores compared with ability scores, and the lack of validity of SRA cutoff scores, how can clinicians use SRAs? Our evidence shows individuals with minimum scores, or slightly above that, are currently at a non- or low-suicidal level and may be treated as such. Individuals with highest or near-highest scores evidence high suicidality/risk and should be treated accordingly. For those scoring in between these extremes, it is not yet possible to determine valid risk groupings. We used S1 data, due to the large volume and diversity on theta, and the suicidal barometer model [27] to help illustrate the suicidal mind (Fig 7). In contrast to CTT-derived cutoff score protocols, and consistent with PROMIS recommendations [80], we propose scores be used to guide but not dictate clinical decisions.

### Limitations and future sustainable scale development

We made efforts to establish valid datasets, however, no treatment of outliers, inauthentic data and missing values can yield perfectly authentic data. Carefully considering these factors resulted in significant improvements over alternatives, such as deleting all cases with missing values or ignoring inauthentic responses [100, 101, 103]. We used cross-sectional convenience samples, which are not ideal but can be as representative of study factor associations, and thus generalizable, as large representative samples [119]. S2 and S3 were moderately-sized but included fewer participants at high suicidality levels, limiting our ability to draw clear conclusions on some model and item characteristics. While studies included sufficient youth samples, we had fewer participants aged 60+. That may be partly due to the online platform. Regardless, further study is required to validate assessment with older ages.

Validating any SRA requires testing prediction abilities. We included a time-two sample (two weeks), however, larger samples over longer periods are required to examine temporal consistency and prediction. To provide more valid tests of SRA predictive abilities, we also require improved measurement of outcomes. We hypothesize that polytomous representations of suicidal outcomes (e.g., suicides, suicide attempts), would provide more information and greater validity than current dichotomous taxa. Several studies have shown suicide attempt status (yes/no) is a demonstrably false dichotomy, as there are degrees of risk within and overlapping taxon [15, 40, 41, 120]. Expanding not-fit-for-purpose categorizations, including cause of death, to a limited continuum can be accomplished through assessing variations in intent to die. For example, Tabachnick [121] and Shneidman [115] promoted the concept of subintentional death, a death that may be due to non-suicidal causes but the decedent was experiencing suicidal symptoms and knowingly put themselves at risk. That and other approaches could help improve the validity of outcome variables for testing SRA predictive abilities.

One of our most important aims was testing the validity of adolescent assessments. Our results fit with previous findings showing children over 12 years are capable of completing self-report psychological assessments [122]. We saw no evidence that younger ages answered discrete visual analogue scales differently than others, and there was no DIF with those aged 13–18. There were no adverse incidents reported, and many adolescents left positive comments regarding their study participation. These findings demonstrate suitability for including youth participants in ethics-approved studies with SRAs, without parental consent. Their volunteer contributions should inspire more efforts to include young people in citizen science type efforts.

There has never been any doubt that valid SRAs can be useful in genetics research, CAT, ecological momentary assessments, etc. Employing low validity measures, however, provides no real benefits with such advanced and potentially groundbreaking approaches. We envision a near future where mental health checkups include CAT using highly validated instruments. These assessments can highlight personal attributes on a network of mental health factors (e.g., depression, suicidality, emotional stability). That information may be combined with neuroimaging techniques, producing more comprehensive psychobiological mental health reports [e.g., 123]. In addition, network analysis has demonstrated unique abilities in describing complex mental health patterns [124]. That, and using precise measurement may help further elucidate the suicidal mind, leading to more insightful work with neuroimaging and genetics. Such a symbiotic mesh of highly valid latent trait and biological evaluation has potential for providing as accurate a picture of mental health as we can for physical health.

In this study, we attempted to conduct scale development according to evidence-based practices, using appropriate measurement models and critically evaluating findings [5, 6, 20]. Sustainable scale development also includes best practices in all areas of psychological science, as well as community involvement. To improve research and clinical practices, we join others in providing publicly available measures [11]. We chose a Creative Commons CC BY 4.0 license for the SS to encourage collaboration and incremental improvements. The SS manual also has a CC BY 4.0 license and will be updated in response to future developments, including SS versions in Chinese, Spanish, etc. [125]. We welcome the suggestions from Kirtley and colleagues [126] on open science in suicidology. We hope such efforts will encourage evidence-based and critical analytic approaches with large datasets, community involvement and using free and open clinical and research instruments.

## Conclusions

For decades, suicide risk assessments have been consistently poor to mediocre. To address this long-standing limitation, we chose a sustainable evidence-driven method to produce a valid and reliable measure–the Suicidality Scale 1.0. It is not perfect. It is, however, a step forward. The SS showed stronger psychometric properties than three comparison scales and demonstrated validity across diverse samples and groups. Using more precise measurement will help elucidate latent traits and refine our psychobiological models. Creating more accurate and sustainable instruments should also translate into improved epidemiology, clinical decisions, and prevention of deaths by despair. If we are to make meaningful inroads into solving the great psychological problems of our times, instrument consistency cannot be allowed to trump measurement validity.

## Supporting information

S1 File (DOCX)

## References

[1] Shanahan L, Hill SN, Gaydosh LM, Steinhoff A, Costello EJ, Dodge KA, et al. Does despair really kill? A roadmap for an evidence-based answer. Am J Public Health. 2019;109(6):854–8. Epub 2019/04/18. doi: 10.2105/AJPH.2019.305016 . 30998413 PMC6506367

[2] Walsh D, McCartney G, Minton J, Parkinson J, Shipton D, Whyte B. Deaths from ’diseases of despair’ in Britain: Comparing suicide, alcohol-related and drug-related mortality for birth cohorts in Scotland, England and Wales, and selected cities. J Epidemiol Community Health. 2021. doi: 10.1136/jech-2020-216220 34045325 PMC8588300

[3] Hawton K, van Heeringen K. Suicide. The Lancet. 2009;373(9672):1372–81. doi: 10.1016/S0140-6736(09)60372-X 19376453

[4] Boudreaux ED, Camargo CA, Arias SA Jr., Sullivan AF, Allen MH, Goldstein AB, et al. Improving suicide risk screening and detection in the emergency department. Am J Prev Med. 2016;50(4):445–53. doi: 10.1016/j.amepre.2015.09.029 26654691 PMC4801719

[5] Borsboom D, Mellenbergh GJ, Van Heerden J. The concept of validity. Psychol Rev. 2004;111(4):1061–71. doi: 10.1037/0033-295X.111.4.1061 PubMed Central PMCID: PMC15482073. 15482073

[6] Flake JK, Fried EI. Measurement schmeasurement: Questionable measurement practices and how to avoid them. Advances in Methods and Practices in Psychological Science. 2020;3(4):456–65. doi: 10.1177/2515245920952393

[7] Cronbach LJ, Shavelson RJ. My current thoughts on coefficient alpha and successor procedures. Educ Psychol Meas. 2004;64(3):391–418. doi: 10.1177/0013164404266386

[8] Sijtsma K, Pfadt JM. Part II: On the use, the misuse, and the very limited usefulness of Cronbach’s alpha: Discussing lower bounds and correlated errors. Psychometrika. 2021. doi: 10.1007/s11336-021-09789-8 34387809 PMC8636457

[9] Nye CD, Drasgow F. Assessing goodness of fit: Simple rules of thumb simply do not work. Org Res Methods. 2011;14(3):548–70. doi: 10.1177/1094428110368562

[10] Hussey I, Hughes S. Hidden invalidity among 15 commonly used measures in social and personality psychology. Advances in Methods and Practices in Psychological Science. 2020;3(2):166–84. doi: 10.1177/2515245919882903

[11] Tackett JL, Brandes CM, King KM, Markon KE. Psychology’s replication crisis and clinical psychological science. Annu Rev Clin Psychol: Annual Reviews Inc.; 2019. p. 579–604. doi: 10.1146/annurev-clinpsy-050718-095710 30673512

[12] Makel MC, Plucker JA, Hegarty B. Replications in psychology research: How often do they really occur? Perspect Psychol Sci. 2012;7(6):537–42. doi: 10.1177/1745691612460688 26168110

[13] Borsboom D. The attack of the psychometricians. Psychometrika. 2006;71(3):425–40. Epub 2006/09/23. doi: 10.1007/s11336-006-1447-6 . 19946599 PMC2779444

[14] Flake JK, Pek J, Hehman E. Construct validation in social and personality research: Current practice and recommendations. Soc Psychol Personal Sci. 2017;8(4):370–8. doi: 10.1177/1948550617693063

[15] Beck AT, Kovacs M, Weissman A. Assessment of suicidal intention: The Scale for Suicide Ideation. J Consult Clin Psychol. 1979;47(2):343–52. doi: 10.1037//0022-006x.47.2.343 469082

[16] Shneidman ES. The suicidal mind. In: Maris RW, Silverman MM, Canetto SS, editors. Review of suicidology, 1997. New York, NY: Guilford Press; 1997. p. 22–41.

[17] Neuringer C, Lettieri DJ. Cognition, attitude, and affect in suicidal individuals. Life Threat Behav. 1971;1(2):106–24.

[18] Raykov T. Estimation of composite reliability for congeneric measures. Appl Psychol Meas. 1997;21(2):173–84.

[19] Graham JM. Congeneric and (essentially) tau-equivalent estimates of score reliability: What they are and how to use them. Educ Psychol Meas. 2006;66(6):930–44. doi: 10.1177/0013164406288165

[20] McNeish D, Wolf MG. Thinking twice about sum scores. Behav Res Methods. 2020;52(6):2287–305. doi: 10.3758/s13428-020-01398-0 32323277

[21] Fried EI, Nesse RM. Depression sum-scores don’t add up: Why analyzing specific depression symptoms is essential. BMC Med. 2015;13(1):72. doi: 10.1186/s12916-015-0325-4 25879936 PMC4386095

[22] Sijtsma K. On the use, the misuse, and the very limited usefulness of Cronbach’s alpha. Psychometrika. 2009;74(1):107–20. doi: 10.1007/s11336-008-9101-0 20037639 PMC2792363

[23] Raykov T. On the use of confirmatory factor analysis in personality research. Pers Individ Dif. 1998;24(2):291–3. doi: 10.1016/S0191-8869(97)00159-1

[24] Rutjes AWS, Reitsma JB, Coomarasamy A, Khan KS, Bossuyt PMM. Evaluation of diagnostic tests when there is no gold standard. A review of methods. Health Technol Assess. 2007;11(50):iii–47. doi: 10.3310/hta11500 PubMed Central PMCID: PMC18021577. 18021577

[25] de Beurs DP, De Vries ALM, De Groot MH, De Keijser J, Kerkhof AJFM. Applying computer adaptive testing to optimize online assessment of suicidal behavior: A simulation study. J Med Internet Res. 2014;16(9). doi: 10.2196/jmir.3511 25213259 PMC4180339

[26] Beck AT, Steer RA, Ranieri WF. Scale for Suicide Ideation: Psychometric properties of a self-report version. J Clin Psychol. 1988;44(4):499–505. doi: 10.1002/1097-4679(198807)44:4&lt;499::aid-jclp2270440404&gt;3.0.co;2-6 3170753

[27] Harris KM, Syu JJ, Lello OD, Chew YLE, Willcox CH, Ho RHM. The ABC’s of suicide risk assessment: Applying a tripartite approach to individual evaluations. PLoS One. 2015;10(6: e0127442). doi: 10.1371/journal.pone.0127442 26030590 PMC4452484

[28] Gibbons RD, Kupfer D, Frank E, Moore T, Beiser DG, Boudreaux ED. Development of a computerized adaptive test suicide scale—The CAT-SS. J Clin Psychiatry. 2017;78(9):1376–82. doi: 10.4088/JCP.16m10922 PubMed Central PMCID: PMC28493655. 28493655 PMC11986876

[29] MacKenzie SB, Podsakoff PM, Jarvis CB. The problem of measurement model misspecification in behavioral and organizational research and some recommended solutions. J Appl Psychol. 2005;90(4):710–30. doi: 10.1037/0021-9010.90.4.710 PubMed Central PMCID: PMC16060788. 16060788

[30] Steeg S, Quinlivan L, Nowland R, Carroll R, Casey D, Clements C, et al. Accuracy of risk scales for predicting repeat self-harm and suicide: A multicentre, population-level cohort study using routine clinical data. BMC Psychiatry. 2018;18(1). doi: 10.1186/s12888-018-1693-z PubMed Central PMCID: PMC29699523. 29699523 PMC5921289

[31] Holden C. Experts map the terrain of mood disorders. Science. 2010;327(5969):1068. 10.1126/science.327.5969.1068-a 20185697

[32] Kendler KS. The dappled nature of causes of psychiatric illness: Replacing the organic-functional/hardware-software dichotomy with empirically based pluralism. Mol Psychiatry. 2012;17(4):377–88. doi: 10.1038/mp.2011.182 22230881 PMC3312951

[33] MacCallum RC, Zhang S, Preacher KJ, Rucker DD. On the practice of dichotomization of quantitative variables. Psychol Methods. 2002;7(1):19–40. doi: 10.1037/1082-989x.7.1.19 11928888

[34] Streiner DL. Breaking up is hard to do: The heartbreak of dichotomizing continuous data. Canadian Journal of Psychiatry. 2002;47(3):262–6. doi: 10.1177/070674370204700307 11987478

[35] Kessler RC, Barker PR, Colpe LJ, Epstein JF, Gfroerer JC, Hiripi E, et al. Screening for serious mental illness in the general population. Arch Gen Psychiatry. 2003;60(2):184–9. doi: 10.1001/archpsyc.60.2.184 PubMed Central PMCID: PMC12578436. 12578436

[36] Kessler RC, Calabrese JR, Farley PA, Gruber MJ, Jewell MA, Katon W, et al. Composite International Diagnostic Interview screening scales for DSM-IV anxiety and mood disorders. Psychol Med. 2013;43(8):1625–37. doi: 10.1017/S0033291712002334 PubMed Central PMCID: PMC23075829. 23075829

[37] Posner K, Brown GK, Stanley B, Brent DA, Yershova KV, Oquendo MA, et al. The Columbia-Suicide Severity Rating Scale: Initial validity and internal consistency findings from three multisite studies with adolescents and adults. Am J Psychiatry. 2011;168(12):1266–77. doi: 10.1176/appi.ajp.2011.10111704 22193671 PMC3893686

[38] Hamilton CM, Strader LC, Pratt JG, Maiese D, Hendershot T, Kwok RK, et al. The PhenX Toolkit: Get the most from your measures. Am J Epidemiol. 2011;174(3):253–60. doi: 10.1093/aje/kwr193 PubMed Central PMCID: PMC21749974. 21749974 PMC3141081

[39] Pek J, Flora DB. Reporting effect sizes in original psychological research: A discussion and tutorial. Psychol Methods. 2018;23(2):208–25. doi: 10.1037/met0000126 PubMed Central PMCID: PMC28277690. 28277690

[40] Harris KM, Lello OD, Willcox CH. Reevaluating suicidal behaviors: Comparing assessment methods to improve risk evaluations. J Psychopathol Behav Assess. 2017;39(1):128–39. doi: 10.1007/s10862-016-9566-6

[41] Harriss L, Hawton K, Zahl D. Value of measuring suicidal intent in the assessment of people attending hospital following self-poisoning or self-injury. Br J Psychiatry. 2005;186(1):60–6. doi: 10.1192/bjp.186.1.60 15630125

[42] DeVellis RF. Scale development: Theory and applications. 4th ed. Bickman L, Rog DJ, editors. Los Angeles, CA: Sage; 2016.

[43] Gleser GC, Cronbach LJ, Rajaratnam N. Generalizability of scores influenced by multiple sources of variance. Psychometrika. 1965;30(4):395–418. doi: 10.1007/BF02289531 5217607

[44] Mair P. Modern psychometrics with R. Gentleman R, Hornik K, Parmigiani G, editors: Springer; 2018.

[45] Reeve BB, Hays RD, Bjorner JB, Cook KF, Crane PK, Teresi JA, et al. Psychometric evaluation and calibration of health-related quality of life item banks: Plans for the Patient-Reported Outcomes Measurement Information System (PROMIS). Med Care. 2007;45(5 SUPPL. 1):S22–S31. doi: 10.1097/01.mlr.0000250483.85507.04 PubMed Central PMCID: PMC17443115. 17443115

[46] UN General Assembly. Resolution Adopted by the General Assembly on 25 September 2015. Transforming our world: The 2030 agenda for sustainable development. United Nations, 2015 Resolution 70/1.

[47] Harris KM, Wang L. The suicidal Mind mapping project. Open Science Framework. 2022. https://osf.io/ac6ng/.

[48] Harris KM, Wang L, Mu GM, Lu Y, So C, Zhang W, et al. Measuring the suicidal mind: The ’open source’ Suicidality Scale, for adolescents and adults. Preprint. 2022. doi: 10.31219/osf.io/b4qut PMC9949661 36821531

[49] Scale validation analyses: The Suicidality Scale development studies [Internet]. figshare. 2022 [cited July 28, 2022].

[50] Décieux JP, Mergener A, Neufang KM, Sischka P. Implementation of the forced answering option within online surveys: Do higher item response rates come at the expense of participation and answer quality? Psihologija. 2015;48(4):311–26. doi: 10.2298/PSI1504311D

[51] Stieger S, Reips UD, Voracek M. Forced-response in online surveys: Bias from reactance and an increase in sex-specific dropout. J Am Soc Inf Sci Technol. 2007;58(11):1653–60. doi: 10.1002/asi.20651

[52] World Medical Association. World Medical Association Declaration of Helsinki: Ethical principles for medical research involving human subjects. J Am Med Assoc. 2013;310(20):2191–4. Epub 2013/10/22. doi: 10.1001/jama.2013.281053 . 24141714

[53] Powell MA, Smith AB. Children’s participation rights in research. Childhood. 2009;16(1):124–42. doi: 10.1177/0907568208101694

[54] Collins TM, Jamieson L, Wright LHV, Rizzini I, Mayhew A, Narang J, et al. Involving child and youth advisors in academic research about child participation: The Child and Youth Advisory Committees of the International and Canadian Child Rights Partnership. Child Youth Serv Rev. 2020;109. doi: 10.1016/j.childyouth.2019.104569

[55] Hanel PHP, Vione KC. Do student samples provide an accurate estimate of the general public? PLoS One. 2016;11(12). doi: 10.1371/journal.pone.0168354 PubMed Central PMCID: PMC28002494. 28002494 PMC5176168

[56] Bentley JP, Thacker PG. The influence of risk and monetary payment on the research participation decision making process. J Med Ethics. 2004;30(3):293–8. doi: 10.1136/jme.2002.001594 PubMed Central PMCID: PMC15173366. 15173366 PMC1733848

[57] R Core Team. R: A language and environment for statistical computing. 4.2.2 ed: R Foundation for Statistical Computing; 2022.

[58] Wang L, Harris KM, Mu GM, Lu Y, So C, Ma J, et al. The Chinese Suicidality Scale: Development and validation of an open instrument for adolescents and adults. Under review. 2023.

[59] Gnambs T, Kaspar K. Disclosure of sensitive behaviors across self-administered survey modes: A meta-analysis. Behav Res Methods. 2014;47(4):1237–59. doi: 10.3758/s13428-014-0533-4 PubMed Central PMCID: PMC25410404. 25410404

[60] Joinson AN, Woodley A, Reips U-D. Personalization, authentication and self-disclosure in self-administered Internet surveys. Comput Human Behav. 2007;23(1):275–85.

[61] Kreuter F, Presser S, Tourangeau R. Social desirability bias in CATI, IVR, and web surveys: The effects of mode and question sensitivity. Public Opin Q. 2008;72(5):847–65. doi: 10.1093/poq/nfn063

[62] Tourangeau R, Yan T. Sensitive questions in surveys. Psychol Bull. 2007;133(5):859–83. doi: 10.1037/0033-2909.133.5.859 17723033

[63] Harris KM, Starcevic V, Ma J, Zhang W, Aboujaoude E. Suicidality, psychopathology, and the internet: Online time vs. online behaviors. Psychiatry Res. 2017;255:341–6. doi: 10.1016/j.psychres.2017.06.012 28601719

[64] Vicente P, Reis E. Using questionnaire design to fight nonresponse bias in web surveys. Soc Sci Comput Rev. 2010;28(2):251–67. doi: 10.1177/0894439309340751

[65] Ware JE, Gandek B. The SF-36 health survey: Development and use in mental health research and the IQOLA project. Int J Ment Health. 1994;23(2):49–73. 10.1080/00207411.1994.11449283.

[66] Revelle W. psych: Procedures for psychological, psychometric, and personality research. [R package version 3.6.2]. In press 2022.

[67] Flora DB, LaBrish C, Chalmers RP. Old and new ideas for data screening and assumption testing for exploratory and confirmatory factor analysis. Front Psychol. 2012;3(MAR). doi: 10.3389/fpsyg.2012.00055 22403561 PMC3290828

[68] Comrey AL, Lee HB. A first course in factor analysis. Hillsdale, NJ: Erlbaum; 1992.

[69] MacCallum RC, Widaman KF, Preacher KJ, Hong S. Sample size in factor analysis: The role of model error. Multivariate Behav Res. 2001;36(4):611–37. doi: 10.1207/S15327906MBR3604_06 26822184

[70] Xia Y, Yang Y. RMSEA, CFI, and TLI in structural equation modeling with ordered categorical data: The story they tell depends on the estimation methods. Behav Res Methods. 2019;51(1):409–28. doi: 10.3758/s13428-018-1055-2 29869222

[71] Brosseau-Liard PE, Savalei V. Adjusting incremental fit indices for nonnormality. Multivariate Behav Res. 2014;49(5):460–70. doi: 10.1080/00273171.2014.933697 26732359

[72] Zhang Z, Yuan K-H. Robust coefficients alpha and omega and confidence intervals with outlying observations and missing data: Methods and software. Educ Psychol Meas. 2016;76(3):387–411. doi: 10.1177/0013164415594658 . 29795870 PMC5965561

[73] Revelle W, Zinbarg RE. Coefficients alpha, beta, omega, and the glb: Comments on Sijtsma. Psychometrika. 2009;74(1):145–54. doi: 10.1007/s11336-008-9102-z

[74] Zinbarg RE, Revelle W, Yovel I, Li W. Cronbach’s α, Revelle’s β and McDonald’s ω H: Their relations with each other and two alternative conceptualizations of reliability. Psychometrika. 2005;70(1):123–33. doi: 10.1007/s11336-003-0974-7

[75] Diener E, Emmons RA, Larsen RJ, Griffin S. The Satisfaction With Life Scale. J Pers Assess. 1985;49(1):71–5. doi: 10.1207/s15327752jpa4901_13 16367493

[76] Kroenke K, Strine TW, Spitzer RL, Williams JBW, Berry JT, Mokdad AH. The PHQ-8 as a measure of current depression in the general population. J Affect Disord. 2009;114(1–3):163–73. doi: 10.1016/j.jad.2008.06.026 PubMed Central PMCID: PMC18752852. 18752852

[77] Kroenke K, Spitzer RL, Williams JBW. The PHQ-9: Validity of a brief depression severity measure. J Gen Intern Med. 2001;16(9):606–13. doi: 10.1046/j.1525-1497.2001.016009606.x 11556941 PMC1495268

[78] Lovibond PF, Lovibond SH. The structure of negative emotional states: Comparison of the Depression Anxiety Stress Scales (DASS) with the Beck Depression and Anxiety Inventories. Behav Res Ther. 1995;33(3):335–43. doi: 10.1016/0005-7967(94)00075-u 7726811

[79] Zimet GD, Dahlem NW, Zimet SG, Farley GK. The Multidimensional Scale of Perceived Social Support. J Pers Assess. 1988;52(1):30–41. 10.1080/00223891.1990.9674095 2280326

[80] PROMIS. PROMIS item bank v2.0, Emotional Support Short Form 6a: PROMIS Health Organization and PROMIS Cooperative Group; 2016. Available from: https://www.healthmeasures.net/index.php?Itemid=992.

[81] Pilkonis PA, Choi SW, Reise SP, Stover AM, Riley WT, Cella D. Item banks for measuring emotional distress from the patient-reported outcomes measurement information system (PROMIS®): Depression, anxiety, and anger. Assessment. 2011;18(3):263–83. doi: 10.1177/1073191111411667 21697139 PMC3153635

[82] U.S. Department of Health and Human Services. Guidance for industry suicidal ideation and behavior: Prospective assessment of occurrence in clinical trials. In: Administration FaD, editor.: Author; 2012.

[83] Hamilton M. Development of a rating scale for primary depressive illness. The British Journal of Social and Clinical Psychology. 1967;6(4):278–96. doi: 10.1111/j.2044-8260.1967.tb00530.x 6080235

[84] Rush AJ, Trivedi MH, Ibrahim HM, Carmody TJ, Arnow B, Klein DN, et al. The 16-item Quick Inventory of Depressive Symptomatology (QIDS), clinician rating (QIDS-C), and self-report (QIDS-SR): A psychometric evaluation in patients with chronic major depression. Biol Psychiatry. 2003;54(5):573–83. doi: 10.1016/s0006-3223(02)01866-8 PubMed Central PMCID: PMC12946886. 12946886

[85] Irwig MS. Depressive symptoms and suicidal thoughts among former users of finasteride with persistent sexual side effects. J Clin Psychiatry. 2012;73(9):1220–3. doi: 10.4088/JCP.12m07887 PubMed Central PMCID: PMC22939118. 22939118

[86] Szanto K, Mulsant BH, Houck P, Dew MA, Reynolds Iii CF. Occurrence and course of suicidality during short-term treatment of late-life depression. Arch Gen Psychiatry. 2003;60(6):610–7. doi: 10.1001/archpsyc.60.6.610 PubMed Central PMCID: PMC12796224. 12796224

[87] Uebelacker LA, German NM, Gaudiano BA, Miller IW. Patient Health Questionnaire depression scale as a suicide screening instrument in depressed primary care patients: A cross-sectional study. Prim Care Companion J Clin Psychiatry. 2011;13(1):e1–e6. doi: 10.4088/PCC.10m01027 21731830 PMC3121214

[88] Hofmans J, Theuns P, Mairesse O. Impact of the number of response categories on linearity and sensitivity of self-anchoring scales: A functional measurement approach. Methodol. 2007;3(4):160–9. doi: 10.1027/1614-2241.3.4.160

[89] Lozano LM, García-Cueto E, Muñiz J. Effect of the number of response categories on the reliability and validity of rating scales. Methodol. 2008;4(2):73–9. doi: 10.1027/1614-2241.4.2.73

[90] Rodriguez A, Reise SP, Haviland MG. Applying bifactor statistical indices in the evaluation of psychological measures. J Pers Assess. 2016;98(3):223–37. doi: 10.1080/00223891.2015.1089249 PubMed Central PMCID: PMC26514921. 26514921

[91] Garrido LE, Abad FJ, Ponsoda V. Are fit indices really fit to estimate the number of factors with categorical variables? Some cautionary findings via monte carlo simulation. Psychol Methods. 2016;21(1):93–111. doi: 10.1037/met0000064 PubMed Central PMCID: PMC26651983. 26651983

[92] Meijer RR, Egberink IJL. Investigating invariant item ordering in personality and clinical scales: Some empirical findings and a discussion. Educ Psychol Meas. 2012;72(4):589–607. doi: 10.1177/0013164411429344

[93] Rizopoulos D. ltm: An R package for latent variable modelling and item response theory analyses. Journal of Statistical Software. 2006;17(5):1–25.

[94] McDonald RP. Test theory: A unified treatment. Mahwah, NJ: Erlbaum; 1999.

[95] Mansolf M, Reise SP. Exploratory bifactor analysis: The Schmid-Leiman orthogonalization and Jennrich-Bentler analytic rotations. Multivariate Behav Res. 2016;51(5):698–717. doi: 10.1080/00273171.2016.1215898 PubMed Central PMCID: PMC27612521. 27612521 PMC5425103

[96] Samejima F. Graded response model. In: Kempf-Leonard K, editor. Encyclopedia of Social Measurement. New York: Academic Press; 2004. p. 77–82.

[97] Black L, Mansfield R, Panayiotou M. Age appropriateness of the self-report Strengths and Difficulties Questionnaire. Assessment. 2020. doi: 10.1177/1073191120903382 32054314

[98] Meade AW, Lautenschlager GJ. A comparison of item response theory and confirmatory factor analytic methodologies for establishing measurement equivalence/invariance. Org Res Methods. 2004;7(4):361–88. doi: 10.1177/1094428104268027

[99] Choi SW, Gibbons LE, Crane PK. lordif: An R package for detecting differential item functioning using iterative hybrid ordinal logistic regression/item response theory and Monte Carlo simulations. Journal of Statistical Software. 2011;39(8):1–30. doi: 10.18637/jss.v039.i08 21572908 PMC3093114

[100] Curran PG. Methods for the detection of carelessly invalid responses in survey data. J Exp Soc Psychol. 2016;66:4–19. doi: 10.1016/j.jesp.2015.07.006

[101] Dupuis M, Meier E, Cuneo F. Detecting computer-generated random responding in questionnaire-based data: A comparison of seven indices. Behav Res Methods. 2019;51(5):2228–37. doi: 10.3758/s13428-018-1103-y 30091086

[102] Huang JL, Liu M, Bowling NA. Insufficient effort responding: Examining an insidious confound in survey data. J Appl Psychol. 2015;100(3):828–45. doi: 10.1037/a0038510 PubMed Central PMCID: PMC25495093. 25495093

[103] Niessen ASM, Meijer RR, Tendeiro JN. Detecting careless respondents in web-based questionnaires: Which method to use? J Res Pers. 2016;63:1–11. doi: 10.1016/j.jrp.2016.04.010

[104] Yentes RD, Wilhelm F. Careless: Procedures for computing indices of careless responding. 1.1.3 ed2018.

[105] Bernaards CA, Sijtsma K. Factor analysis of multidimensional polytomous item response data suffering from ignorable item nonresponse. Multivariate Behav Res. 1999;34(3):277–313. doi: 10.1207/S15327906MBR3403_1

[106] Dong Y, Peng CYJ. Principled missing data methods for researchers. SpringerPlus. 2013;2(1):1–17. doi: 10.1186/2193-1801-2-222 23853744 PMC3701793

[107] Efron B. Missing data, imputation, and the bootstrap. J Am Stat Assoc. 1994;89(426):463–75. doi: 10.1080/01621459.1994.10476768

[108] Bland JM, Altman DG. Statistics notes: Bootstrap resampling methods. BMJ (Online). 2015;350. doi: 10.1136/bmj.h2622 PubMed Central PMCID: PMC26037412. 26037412

[109] Reise SP, Moore TM, Haviland MG. Bifactor models and rotations: Exploring the extent to which multidimensional data yield univocal scale scores. J Pers Assess. 2010;92(6):544–59. doi: 10.1080/00223891.2010.496477 20954056 PMC2981404

[110] Gorsuch RL. Factor analysis. Hillsdale, NJ: Lawrence Erlbaum; 1983.

[111] Carvalho CB, Nunes C, Castilho P, da Motta C, Caldeira S, Pinto-Gouveia J. Mapping non suicidal self-injury in adolescence: Development and confirmatory factor analysis of the impulse, self-harm and suicide ideation questionnaire for adolescents (ISSIQ-A). Psychiatry Res. 2015;227(2–3):238–45. doi: 10.1016/j.psychres.2015.01.031 PubMed Central PMCID: PMC25908263. 25908263

[112] Baumeister RF. Suicide as escape from self. Psychol Rev. 1990;97(1):90–113. doi: 10.1037/0033-295x.97.1.90 2408091

[113] Cummings LR, Mattfeld AT, Pettit JW, McMakin DL. Viewing nonsuicidal self-injury in adolescence through a developmental neuroscience lens: The impact of neural sensitivity to socioaffective pain and reward. Clinical Psychological Science. 2021;9(5):767–90. doi: 10.1177/2167702621989323

[114] Kovacs M, Beck AT. The wish to die and the wish to live in attempted suicides. J Clin Psychol. 1977;33(2):361–5. doi: 10.1002/1097-4679(197704)33:2&lt;361::aid-jclp2270330207&gt;3.0.co;2-h 870525

[115] Shneidman ES. Orientations toward death: Subintentioned death and indirect suicide. Suicide Life Threat Behav. 1981;11(4):232–53. 7330914

[116] Madsen J, Harris KM. Negative self-appraisal: Personal reasons for dying as indicators of suicidality. PLoS One. 2021;16(2):e0246341. doi: 10.1371/journal.pone.0246341 33529221 PMC7853472

[117] Evans G, Farberow NL. The encyclopedia of suicide. New York: Facts on File; 1988.

[118] Neuringer C. Relationship between life and death among individuals of varying levels of suicidality. J Consult Clin Psychol. 1979;47(2):407–8. doi: 10.1037//0022-006x.47.2.407 PubMed Central PMCID: PMC469092. 469092

[119] Mullinix KJ, Leeper TJ, Druckman JN, Freese J. The generalizability of survey experiments. J Exp Political Sci. 2015;2(2):109–38. doi: 10.1017/XPS.2015.19

[120] Suokas J, Suominen K, Isometsaè E, Ostamo A, Loènnqvist J. Long-term risk factors for suicide mortality after attempted suicide—Findings of a 14-year follow-up study. Acta Psychiatr Scand. 2001;104(2):117–21. doi: 10.1034/j.1600-0447.2001.00243.x 11473505

[121] Tabachnick N. Subintentioned self-destruction in teenagers. Psychiatric Opinion. 1975;12(6):21–6.

[122] Matza LS, Patrick DL, Riley AW, Alexander JJ, Rajmil L, Pleil AM, et al. Pediatric patient-reported outcome instruments for research to support medical product labeling: Report of the ISPOR PRO good research practices for the assessment of children and adolescents task force. Value Health. 2013;16(4):461–79. doi: 10.1016/j.jval.2013.04.004 23796280

[123] Husain SF, Yu R, Tang TB, Tam WW, Tran B, Quek TT, et al. Validating a functional near-infrared spectroscopy diagnostic paradigm for Major Depressive Disorder. Sci Rep. 2020;10(1). doi: 10.1038/s41598-020-66784-2 PubMed Central PMCID: PMC32546704. 32546704 PMC7298029

[124] Beard C, Millner AJ, Forgeard MJC, Fried EI, Hsu KJ, Treadway MT, et al. Network analysis of depression and anxiety symptom relationships in a psychiatric sample. Psychol Med. 2016;46(16):3359–69. doi: 10.1017/S0033291716002300 PubMed Central PMCID: PMC27623748. 27623748 PMC5430082

[125] Harris KM, Wang L. The Suicidality Scale manual. OSF Preprints. 2022. doi: 10.31219/osf.io/6tknd

[126] Kirtley OJ, Janssens JJ, Kaurin A. Open Science in Suicide Research Is Open for Business. Crisis. 2022;43(5):355–60. doi: 10.1027/0227-5910/a000859 PubMed Central PMCID: PMC35915973. 35915973 PMC9645435

