# Supplementary material for: Measuring the suicidal mind: The ‘open source’ Suicidality Scale, for adolescents and adults
Source: PLoS One. 2023 Feb 23;18(2):e0282009. doi: 10.1371/journal.pone.0282009 (PMC9949661; doi:10.1371/journal.pone.0282009)
Supplement: S1 File — (DOCX) [file pone.0282009.s001.docx]

**Appendix A**

**Suicidality Item Pool**

| **Item** | **Source** | **Prompt** | **Responses** |
| --- | --- | --- | --- |
| Dead | PHQ-9 | Over the past 2 weeks, how often have you been bothered by any of the following problems? Thoughts that you would be better off dead or of hurting yourself in some way | 0 = Not at all; 1 = Several days; 2 = More than half the days; 3 = Nearly every day |
| Ideation-lifetime | Various | Have you ever thought about killing yourself? | 0 = Never; 1 = Yes, but never seriously thought about dying; 2, 3, 4 = Yes, and at least once seriously though about dying |
| Plan-intent | Various | Have you ever made a plan to kill yourself? | 0 = Never; 1 = Yes, but it wasn’t a serious plan; 2, 3, 4 = Yes, and at least once made a very serious plan |
| Attempt-intent | Various | Have you ever attempted to kill yourself? | 0 = Never; 1 = Yes, but didn’t really want to die; 2, 3, 4 = Yes, and at least really wanted to die |
| Ideation-year | Various | How often have you thought about killing yourself in the past year? | 0 = Never, 2, 3, 4 = Frequently |
| Debate-year | SABCS | In the past year, have you had an internal debate/argument (in your head) about whether to live or die? | 0 = Never, 2, 3, 4 = Frequently |
| WTLr | SSI, SABCS | Recently, how much do you wish to live? | 0 = Not at all, 1, 2, 3, 4, 5, 6 = Very much (reverse-scored) |
| WTD | SSI, SABCS | Recently, how much do you wish to die? | 0 = Not at all, 1, 2, 3, 4, 5, 6 = Very much |
| Predict | SBQ, SABCS | How likely is it that you will attempt suicide someday? | 0 = Not at all, 1, 2, 3,4 = Very likely |
| Wish-HAM-D | HAM-D | Recently, have you had thoughts that life is not worth living, thinking you’d be better off dead, wishing you were dead, thoughts of hurting or killing yourself? | 0 = Not at all; 1 = My life is not worth living; 2 = I wish I were dead, or I think about my death; 3 = I think about suicide, or I told someone I think about suicide; 4 = I have attempted suicide and wanted/tried to die |
| Ideation-BDI | BDI-II | Recently, have you had suicidal thoughts or wishes? | 0 = I don’t have any thoughts of killing myself; 1 = I have thoughts of killing myself, but I would not carry them out; 2 = I would like to kill myself; 3 = I would kill myself if I had the chance |
| RFD | SSI |  | 0 = My reasons for living outweigh my reasons for dying; 2, 3, 4 = My reasons for dying outweigh my reasons for living |
| DKS | SSI |  | 0 = I have no desire to kill myself; 1, 2, 3, 4 = I have a strong desire to kill myself |
| Save | SSI |  | 0 = I would try to save my life if I found myself in a life-threatening situation; 1, 2, 3, 4 = I would not take the steps necessary to avoid death if I found myself in a life-threatening situation |
| Meaning | DASS | Over the past week:  I felt that life was meaningless | 0 = Did not apply to me at all; 1, 2, 3 = Applied to me very much/most of the time |
| Ideation-times | C-SSRS, various | How many times have you thought about killing yourself? | 0 = Never, 1 = Less than once a week; 2 = Once a week, 3 = 2-5 times a week, 4 = Daily or almost daily, 5 = Many times a day |
| Ideation-hours | C-SSRS | When you have suicidal thoughts, how long do they last? | 0 = Never have suicidal thoughts; 1 = Fleeting, - few seconds or minutes; 2 = Less than 1 hour/some of the time, 3 = 1-4 hours/a lot of time, 4 = 4-8 hours/most of the day, 5 = More than 8 hours/persistent or continuous |
| Ideation-control | C-SSRS | Could/can you stop thinking about killing yourself or wanting to die if you want to? | 0 = Easily able to control thoughts; 1 = Can control thoughts with little difficulty; 2 = Can control thoughts with some difficulty; 3 = Can control thoughts with a lot of difficulty; 4 = Unable to control thoughts; 5 = I do not attempt to control thoughts |
| Deterrents | C-SSRS | Are there things – anyone or anything (e.g., family, religion, pain of death) – that stopped you from wanting to die or acting on thoughts of committing suicide? | 0 = Does not apply; 1 = Deterrents definitely stopped you from attempting suicide; 2 = Deterrents probably stopped you; 3 = Uncertain that deterrents stopped you; 4 = Deterrents most likely did not stop you; 5 = Deterrents definitely did not stop you |
| Reasons | C-SSRS | What sort of reasons did you have for thinking about wanting to die or killing yourself? Was it to end the pain or stop the way you were feeling (in other words you couldn’t go on living with this pain or how you were feeling) or was it to get attention, revenge or a reaction from others? Or both? | 0 = Does not apply; 1 = Completely to get attention, revenge or a reaction from others; 2 = Mostly to get attention, revenge or a reaction from others; 3 = Equally to get attention, revenge or a reaction from others and to end/stop the pain; 4 = Mostly to end or stop the pain (you couldn’t go on living with the pain or how you were feeling); 5 = Completely to end or stop the pain (you couldn’t go on living with the pain or how you were feeling) |
| Sleep-b | C-SSRS | Have you wished you were dead or wished you could go to sleep and not wake up? | 0 = No; 1 = Yes |
| Ideation-b | C-SSRS, various | Have you actually had any thoughts of killing yourself? | 0 = No; 1 = Yes |
| How-b | C-SSRS | Have you been thinking about how you might kill yourself? | 0 = No; 1 = Yes |
| Intent-b | C-SSRS | Have you had thoughts of killing yourself and had some intentions of acting on them? | 0 = No; 1 = Yes |
| Plan-b | C-SSRS | Have you started to work out or worked out the details of how to kill yourself? Do you intend to carry out this plan? | 0 = No; 1 = Yes |
| Attempt-b | C-SSRS, various | Have you made a suicide attempt with some intent to die? | 0 = No; 1 = Yes |
| Self-harm-b | C-SSRS | Have you done anything to harm yourself, but with no intent to die? | 0 = No; 1 = Yes |
| Stopped-b | C-SSRS | Has there been a time when you started to do something to end your life but someone or something stopped you before you actually did anything? | 0 = No; 1 = Yes |
| Stop-self-b | C-SSRS | Has there been a time when you started to do something to try to end your life but you stopped yourself before you actually did anything? | 0 = No; 1 = Yes |
| Prepare-b | C-SSRS | Have you done anything, started to do anything, or prepared to do anything to end your life? (examples: collected pills, gave away valuables, wrote a suicide note). | 0 = No; 1 = Yes |

*Note*. SSI = Scale for Suicide Ideation, SBQ = Suicidal Behaviors Questionnaire, SABCS = Suicidal Affect-Behavior-Cognition Scale, C-SSRS = Columbia-Suicide Severity Scale, PHQ-9 = Patient Health Questionnaire, b = binary.

**Appendix B**

**Suicidality Scale Changes Across Studies**

| Item | Study 1 | Responses | Study 2 | Responses | Study 3 | Responses |
| --- | --- | --- | --- | --- | --- | --- |
| Dead | Over the past 2 weeks, how often have you been bothered by any of the following problems?  Thoughts that you would be better off dead or of hurting yourself in some way | 1 = Not at all; 2 = Several days; 3 = More than half the days; 4 = Nearly every day | **Recently**, I have been bothered by thoughts that I would be better off dead | 1 = Never; 5 = Very often | Recently, **have you** been bothered by thoughts that you would be better off dead? | 1 = Never; Very often |
| Debate | **In the past year**, have you had an internal debate/argument (in your head) about whether to live or die? | 1 = Never; 5 = Frequently | Have you had an internal debate/argument (in your head) about whether to live or die? | 1 = Never; 5 = Very often | **Recently**, have you had.. | 1 = Never; 5 = Very often |
| Meaning | Over the **past** **week**:  I felt that life was meaningless | 1 = Did not apply to me at all; 4 = Applied to me very much/most of the time | **Recently**, I have felt my life is meaningless | 1 = Never; 5 = Very often | Recently, **have you** felt your life is meaningless? | 1 = Never; 5 = Very often |
| WTD | **Recently**, how much do you wish to die? | 1 = Not at all; 7 = Very much | How much do you wish to die? | 1 = Not at all; 7 = Very much | **Recently**, how much do you wish to die? | 1 = Not at all; 7 = Very much |
| RFD |  | 1 = My reasons for living outweigh my reasons for dying; 5 = My reasons for dying outweigh my reasons for living |  | 1 = My reasons for living outweigh (**are greater than**) my reasons for dying; 5 = My reasons for dying outweigh (**are greater than**) my reasons for living | 1 = My reasons for living are greater than my reasons for dying; 5 = My reasons for dying are greater than my reasons for living |  |

*Note*. Changed text in **bold**.

**Appendix C**

**The Suicidality Scale 1.0**

**Instructions**: We would like to ask you some personal questions related to killing oneself. Please indicate the response that matches you best.

| **Code** | **Item/prompt** | **Responses** |
| --- | --- | --- |
| Ideation | How often have you thought about killing yourself in the past year? | 1 = Never, 5 = Very often |
| Debate | In the past year, have you had an internal debate/argument (in your head) about whether to live or die? | 1 = Never, 5 = Very often |
| Dead | Recently, have you been bothered by thoughts that you would be better off dead? | 1 = Never, 5 = Very often |
| Meaning | Recently, have you felt your life is meaningless? | 1 = Never, 5 = Very often |
| WTD | Recently, how much do you wish to die? | 1 = Not at all, 5 = Very much |
| Predict | How likely is it that you will attempt suicide someday? | 1 = Not at all, 5 = Very likely |
| RFD |  | 1 = My reasons for living are greater than my reasons for dying, 5 = My reasons for dying are greater than my reasons for living |
| DKS |  | I have no desire to kill myself = 1, I have a strong desire to kill myself |
|  | ***Supplementary items***  *(not included in calculations)* |  |
| WTL* | Recently, how much do you wish to live? | 5 = Not at all, 1 = Very much |
| Attempt | Have you ever attempted to kill yourself? | 1 = Never; 2 = Yes, but never really wanted to die, 3 = Yes, but was uncertain about dying, 4 = Yes, and at least once really wanted to die |
| Plan | Have you ever made a plan to kill yourself? | 1 = Never, 2 = Yes, but never really wanted to die, 3 = Yes, but was uncertain about dying, 4 = Yes, and at least once really wanted to die |

*This item is reverse-scored and should be used cautiously, particularly with non-native English speakers or when translated into other languages.
